# Supplementary material for: Altering metabolism programs cell identity via NAD+-dependent deacetylation
Source: EMBO J. 2025 Apr 25;44(11):3056–84. doi: 10.1038/s44318-025-00417-0 (PMC12130289; doi:10.1038/s44318-025-00417-0)
Supplement: Supplementary file 1 — Appendix [file 44318_2025_417_MOESM1_ESM.pdf]

Appendix File

Table of Contents

Appendix Figure S1: Proteomic and Metabolic Signatures Associated with Distinct ESC Culture Conditions. ………2

Appendix Figure S2: Characterization of the Metabolic and Cell Cycle Characteristics of EMESCs ………4

Appendix Figure S3: Differentiation Potential Characteristics of EMESCs and Effects of Galactose on Pre-Implantation Development. ………………………………………………………………6

Appendix Figure S4: Further Characterization of Acetylome of EMM-cultured ESCs. ……………..8

Appendix Figure S5: Sirt1-KO and Sirt1-FKBP cell line validation. ………………………………………10

References. ………12

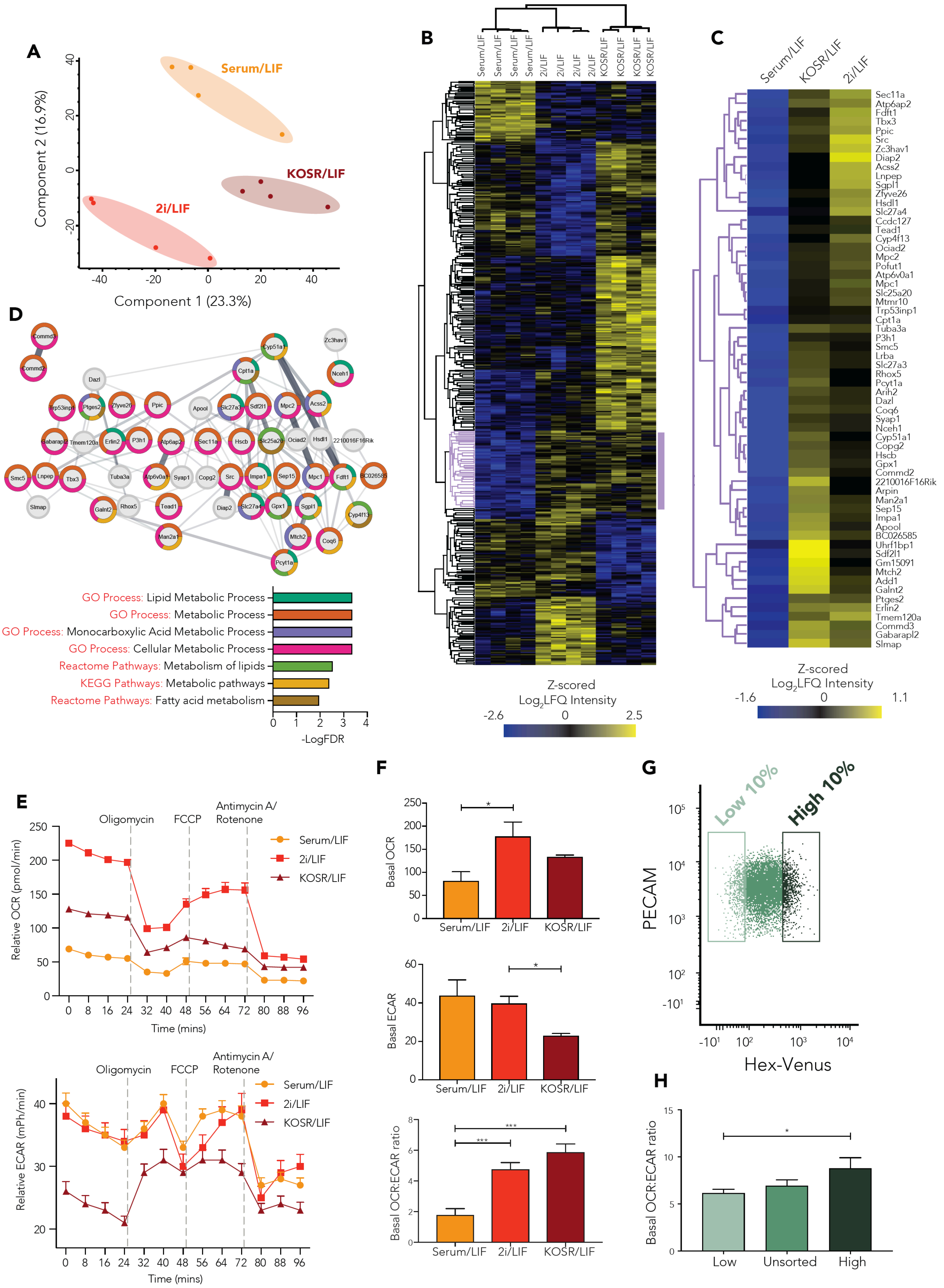

Appendix Figure S1: Proteomic and Metabolic Signatures Associated with Distinct ESC Culture Conditions.

A) PCA analysis of differentially expressed proteins in ESCs cultured in Serum/LIF-, 2i/LIF and KOSR/LIF.

B) Heatmap for differentially expressed proteins (log_2(FC)>1, P_{adj}<0.05) between Serum/LIF-, 2i/LIF- and KOSR/LIF-cultured ESCs after 3 passages, *n*=4 biologically independent samples, see also Supplementary Table 1.

C) Commonly upregulated proteins between 2i/LIF- and KOSR/LIF-cultured ESCs, relative to Serum/LIF-cultured ESCs (purple branch of left panel in (A)).

D) STRING diagram of proteins upregulated in 2i/LIF- and KOSR/LIF-cultured ESCs, relative to Serum/LIF-cultured ESCs (top); Gene Ontology (GO) Biological Processes, Reactome and (Kyoto Encyclopedia of Genes and Genomes (KEGG) pathways table of proteins upregulated in 2i/LIF- and KOSR/LIF-cultured ESCs, relative to Serum/LIF-cultured ESCs (bottom).

E) Graphs depicting full mito-stress test, with addition of 1μM Oligomycin, 3μM FCCP and 1μM Antimycin A/Rotenone at t=25, 49 and 73 mins respectively.

F) Basal OCR (top), ECAR (middle) and OCR:ECAR ratio (bottom) analysis for between Serum/LIF-, 2i/LIF- and KOSR/LIF-cultured ESCs. n=24 technical replicates, from 3 independent experiments. Bar charts depict mean and S.E.M, p-values: OCR Serum/LIF vs 2i/LIF *p=0.0153; ECAR 2i/LIF vs KOSR/LIF *p=0.0182; OCR/ECAR Serum/LIF vs 2i/LIF ***p=0.0008, Serum/LIF vs KOSR/LIF ***p=0.0004; unpaired two-tailed t-**test.**

G) Dot plot showing FACS for populations of Hhex-Venus-sorted ESCs cultured in 2i/LIF, where boxes denote the 10% low and 10% high fractions. Representative of 2 biologically independent samples.

H) Basal OCR:ECAR ratio analysis comparing Hhex-Venus 10% low, unsorted and 10% high fractions of ESCs cultured in 2i/LIF. *n*=10 technical replicates from 2 biologically independent samples. Bar charts depict mean and SEM, p-values: *p=0.0161; unpaired two-tailed t-**test.**

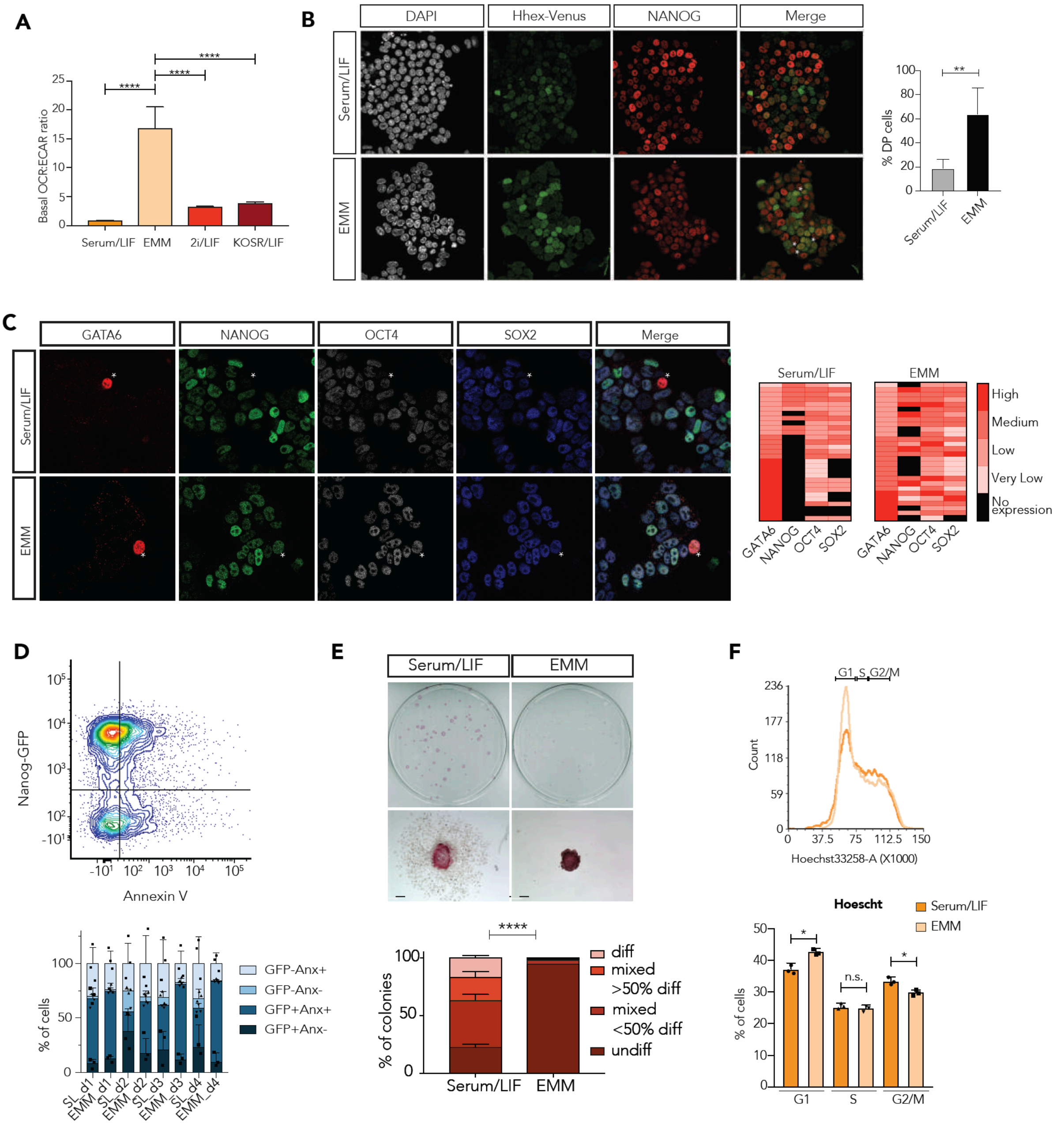

Appendix Figure S2: Characterization of the Metabolic and Cell Cycle Characteristics of EMESCs.

A) Basal OCR:ECAR ratio for ESCs cultured in Serum/LIF, 2i/LIF, and KOSR/LIF for 3 passages, and EMESCs cultured in EMM for 48h. n=24 technical replicates, from 3 independent experiments. Bar chart depicts mean and S.E.M. *****p<0.001, unpaired two-tailed t-test.

B) Hhex-Venus reporter ESCs cultured for 2 passages in Serum/LIF or EMM, then immunostained for NANOG and Hhex-Venus (GFP antibody). White asterisk marks example

cells with co-expression. Quantification of double positive (DP) cells as a percentage of total Hhex-Venus positive cells, from 6 images of 2 biologically independent samples per condition, **p=0.0099, unpaired two-tailed t-test. Scale bar = 20μm.

C) Immunostaining of Serum/LIF-cultured ESCs or EMESCs (48h) for GATA6, NANOG, OCT4 and SOX2, with quantification of 30 individual GATA6$^+$ cells taken from 3 independent experiments. Scale bar = 20μm.

D) Annexin V staining of EMESCs after d1-4 in EMM culture. FACS plot depicts Nanog-eGFP reporter co-expression with Annexin V (representative of 3 independent experiments), with quantification of Annexin V staining in Nanog^{high} and Nanog^{low} gates (gated against GFP-negative cells) at all timepoints.

E) ESC and EMESC colonies from individual cells cultured in Serum/LIF or EMM respectively for 9 days, stained for alkaline phosphatase activity (Representation of colonies from each condition). Scale bar = 800μm. Quantification of ESC colonies from 3 biologically independent samples per condition, ****p <0.0001, two-tailed chi-square test.

F) Cell cycle profiles of Serum/LIF-cultured ESCs and EMESCs after 2 passages, following staining with Hoescht33342 and analyzed by Flow Cytometry. EMESCs displayed increased proportion in G1 phase (37.21% to 42.73%), and decreased proportion in G2/M (38.10% to 31.72%), *p<0.05, unpaired two-tailed t-test.

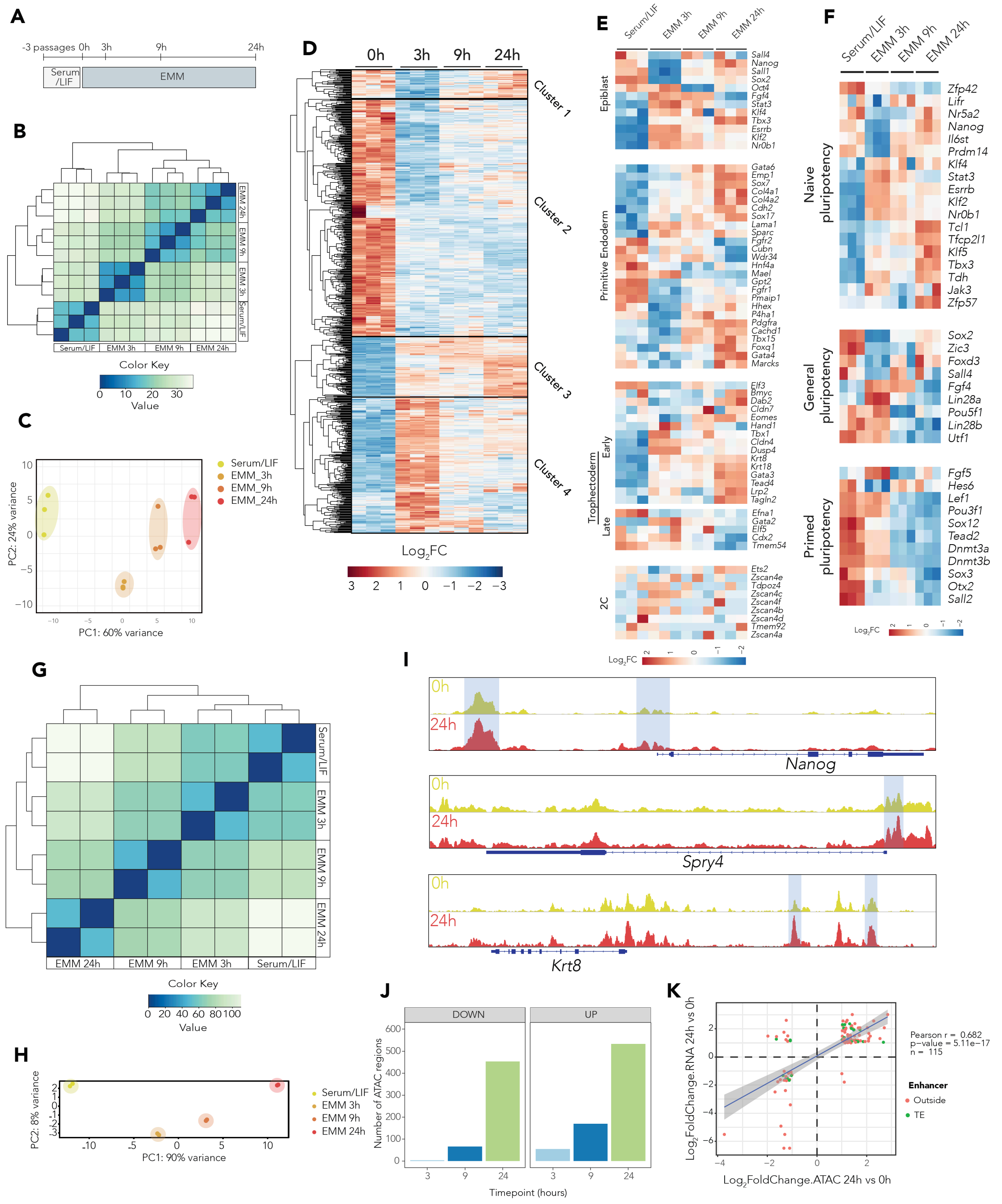

Appendix Figure S3: Early Transcriptional and Chromatin Accessibility Changes in Response to Culture in EMM.

A) Experimental outline for EMM time course used for ATAC- and RNA-seq analysis.

B) Unbiased hierarchical clustering of whole-transcriptome RNA-seq datasets for ESCs cultured in Serum/LIF or EMM for 3h, 9h, and 24h. n=3 experimentally independent samples.

C) PCA for RNAseq of ESCs cultured in Serum/LIF or EMM for 3h, 9h, and 24h.

D) RNA-seq heatmap of differentially expressed genes ($Log_2FC>1$, $P_{adj}<0.01$) observed in ESCs cultured in Serum/LIF or EMM, with 4 clusters.

E) RNA-seq heat map depicting changes in Epi, PrE, TE and 2C marker gene transcription during EMM timecourse.

F) RNA-seq heat map depicting changes in naïve, general, and primed pluripotency marker gene transcription in response to EMM.

G) Unbiased hierarchical clustering of whole-epigenome ATAC-seq datasets for ESCs cultured in Serum/LIF or EMM for 3h, 9h, and 24h. n=2 experimentally independent samples.

H) PCA of ATAC-seq for ESCs cultured in Serum/LIF or EMM for 3h, 9h, and 24h.

I) ATAC-seq profiles of *Nanog*, *Spry4* and *Krt8* genes depicting increased chromatin accessibility at enhancer regions between 0h and 24h of EMM culture, shaded boxes depict EP300 peaks from ChIP-seq data (Hamilton *et al*, 2019).

J) Numbers of significantly up- or down-regulated ($Log_2FC>1$, $P_{adj}<0.01$) ATAC-seq regions relative to Serum/LIF at 3, 9 and 24h EMM culture.

K) Scatterplot depicting changes in RNA expression after 24h EMM, with associated ATAC-regions at 24h EMM ($Log_2FC>1$, $P_{adj}<0.01$). TEs shown as green dots.

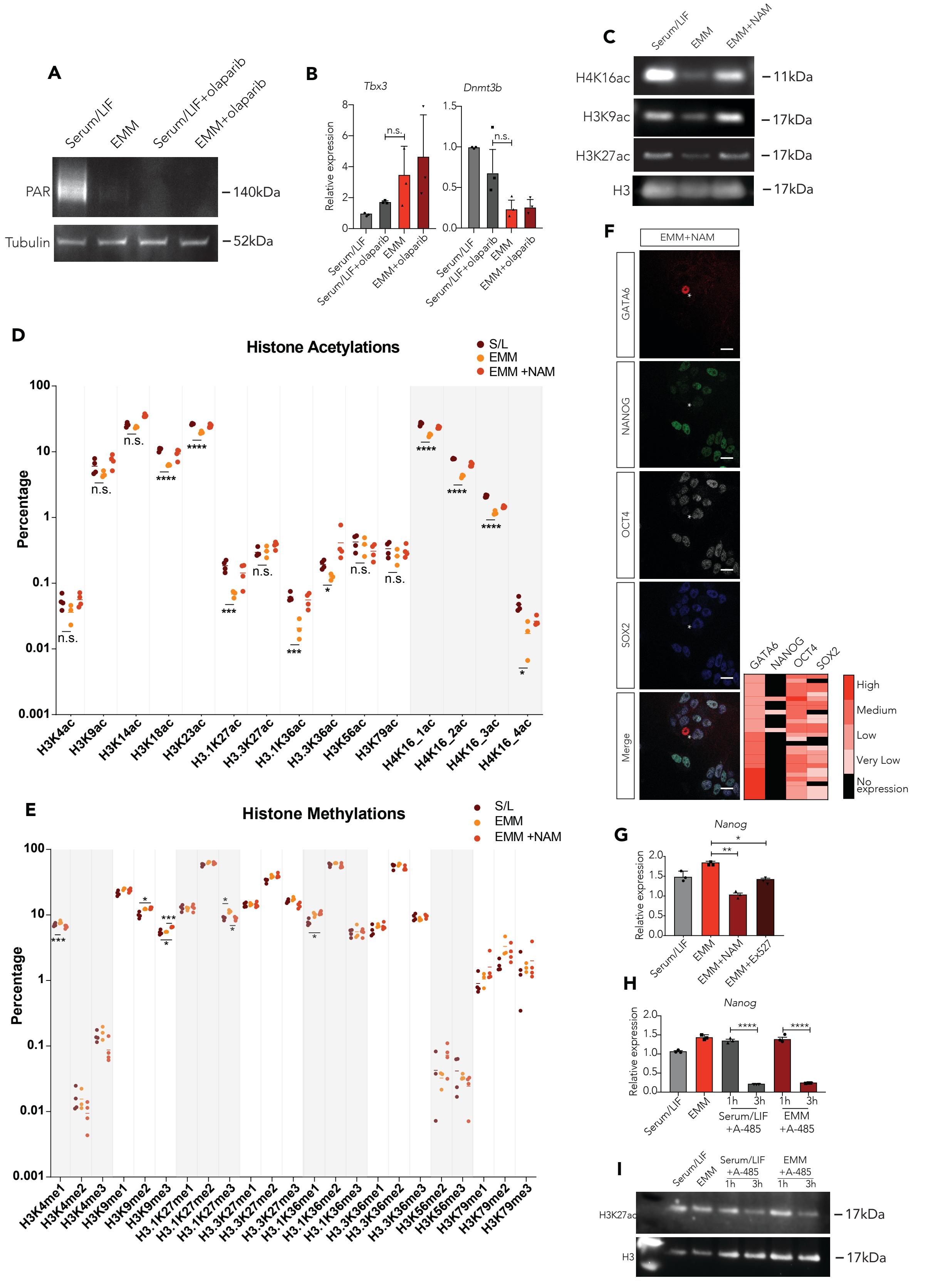

Appendix Figure S4: Further Downstream Response to EMM Metabolic Phenotypes and the Enzymes Propagating them.

A) Western blot analysis of PARP activity (auto-PARylation, left) in ESCs cultured in Serum/LIF-+ or EMESCs cultured in EMM-+ PARP inhibitor Olaparib (10µM) for 24h. Representative of 2 biological replicates.

B) RT-qPCR of EMM-early response genes (*Tbx3* and *Dnmt3b*) in ESCs cultured in Serum/LIF-+ or EMESCs cultured in EMM-+ PARP inhibitor Olaparib (10uM) for 24h. Data are mean + s.d. n=3 biologically independent samples. p-values: *Tbx3* n.s.=0.3186; *Dnmt3b* n.s.=0.1770, unpaired two-tailed t-test.

C) Western blot analysis of protein levels H3K9ac, H3K27ac and H4K16ac levels, relative to total H3 levels in ESCs cultured in Serum/LIF and EMESCs cultured in EMM-/+NAM for 24h. Data are representative of 3 biologically independent samples.

D) Global histone acetylation levels quantified by mass spectrometry. n=4 biological replicates (3 for EMM). Multiple unpaired t-tests. Lines indicate median, boxes represent first and third quartiles and whiskers extend 1.5× IQR. p-values: H3K4ac n.s.=0.1432; H3K9ac n.s.=0.1816; H3K14ac n.s.=0.1091; H3K18ac ****p<0.0001; H3K23ac ****p<0.0001; H3.1K27ac **p=0.0029; H3.3K27ac n.s.=0.2620; H3.1K36ac **p=0.0015; H3.3K36ac *p=0.0132; H3K56ac n.s.=0.6347; H3K79ac n.s.=0.1628; H4K4..17_1ac ****p<0.0001; H4K4..17_2ac ****p<0.0001; H4K4..17_3ac ***p=0.0001; H4K4..17_4ac *p=0.0128.

E) Global histone methylation levels quantified by mass spectrometry. n=4 biological replicates (3 for EMM). Two-way ANOVA. Lines indicate median, boxes represent first and third quartiles and whiskers extend 1.5× IQR. p-values: H3K4me1 ***p=0.0003; H3K9me2 *p=0.0216; H3K9me3 SL vs EMM+NAM *p=0.0216, EMM vs EMM+NAM ***p=0.0003; H3.1K27me3 SL vs EMM *p=0.0368, EMM vs EMM+NAM *p=0.0179; H3.1K36me1 *p=0.0216.

F) Immunostaining of EMM+NAM-cultured (48h) ESCs for GATA6, NANOG, OCT4 and SOX2, with quantification of 30 individual GATA6+ cells taken from 3 independent experiments. Scale bar = 20μm.

G) RT-qPCR analysis of *Nanog* mRNA affected by EMM culture in ESCs cultured in Serum/LIF, and EMESCs cultured in EMM, EMM+NAM (20mM), and Ex-527 (10μM) for 24h. Data are mean + s.d., unpaired two-tailed t-test. p-values: EMM vs EMM+NAM **p=0.0012; EMM vs EMM+10uM Ex527 *p=0.0301.

H) RT-qPCR analysis for *Nanog* mRNA in Serum/LIF-cultured ESCs and EMESCs after 1h and 3h culture -+A-485 (10nM). Data are mean + s.d., ****p<0.0001, unpaired two-tailed t-test.

I) Western blot analysis for and H3K27ac in Serum/LIF-cultured ESCs and EMESCs after 1h and 3h culture -+A-485 (10nM).

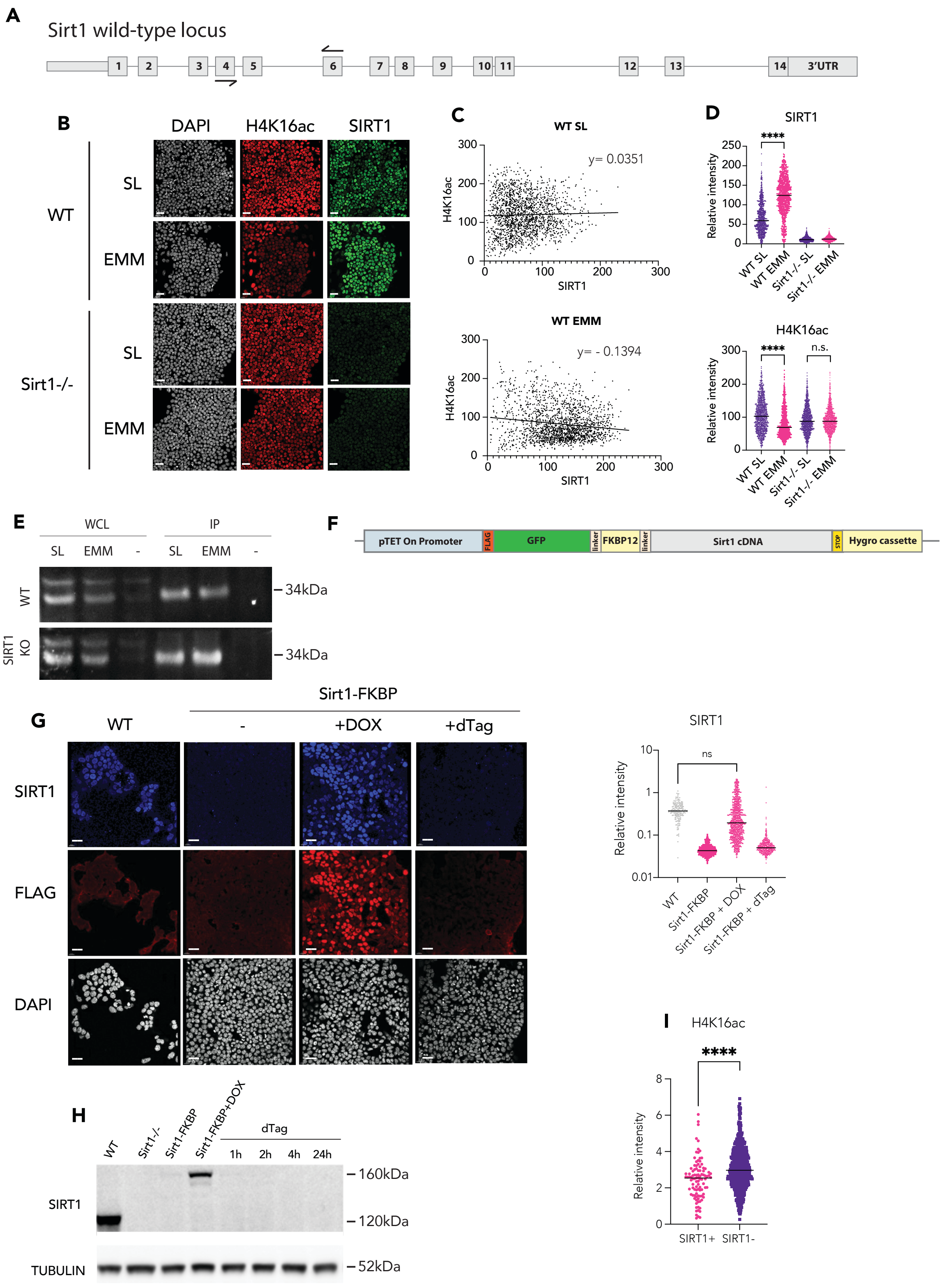

Appendix Figure S5: Sirt1-KO and Sirt1-FKBP cell line validation.

A) Sirt1 endogenous locus, depicting CRISPR guides cutting at exons 4 and 6, used to generate $Sirt1^{-/-}$ KO mESC lines.

B) IF for H4K16ac and SIRT1 in SL- and EMM-cultured ESCs, both for WT and $Sirt1^{-/-}$ background. Scale bar = 30μm.

C) Dot plot depicting correlation between SIRT1 and H4K16ac levels in SL- (top) and EMM- (bottom) cultured ESCs.

D) Quantification of SIRT1 (top) and H4K16ac (bottom) in SL- and EMM-cultured ESCs, both for WT and $Sirt1^{-/-}$ background. Samples from 2 independent experiments, ****p<0.0001, n.s.=0.5101, unpaired two-tailed t-test.

E) IP for Acetylated lysine and Western blot analysis of SOX2 in ESCs cultured for 24h in Serum/LIF and EMM, and IgG control (-) in both WT and in Sirt1-KO ESCs. Data are representative of 3 biologically independent samples.

F) Cartoon of TET On-inducible eGFP-FLAG-Sirt1-FKBP12 construct, used to generate Sirt1-FKBP mESC lines in Sirt1 KO background.

G) IF for FLAG and SIRT1 in WT and Sirt1-FKBP mESCs with addition of DOX (48h) and/ dTag-13 (24h)(left); Quantification of SIRT1 levels in WT mESCs, and in Sirt1-FKBP mESCs -/+DOX, -/+ dTag-13 (right). Samples from 2 independent experiments, n.s.=0.1452, unpaired two tailed t-test. Scale bar = 30μm.

H) Western Blot analysis of SIRT1 expression in WT and Sirt1-FKBP mESCs, with timecourse of dTag-13 addition. Depiction of 3 biologically independent samples.

I) IF Quantification of H4K16ac levels in SIRT1- and SIRT1+ ESCs. Samples from 2 independent experiments, ****p<0.0001, unpaired two-tailed t-test.

References

Hamilton WB, Mosesson Y, Monteiro RS, Emdal KB, Knudsen TE, Francavilla C, Barkai N, Olsen JV & Brickman JM (2019) Dynamic lineage priming is driven via direct enhancer regulation by ERK. *Nature* 575: 355–360
