# Supplementary figures and images for: Altering metabolism programs cell identity via NAD+-dependent deacetylation
Source: EMBO J. 2025 Apr 25;44(11):3056–84. doi: 10.1038/s44318-025-00417-0 (PMC12130289; doi:10.1038/s44318-025-00417-0)

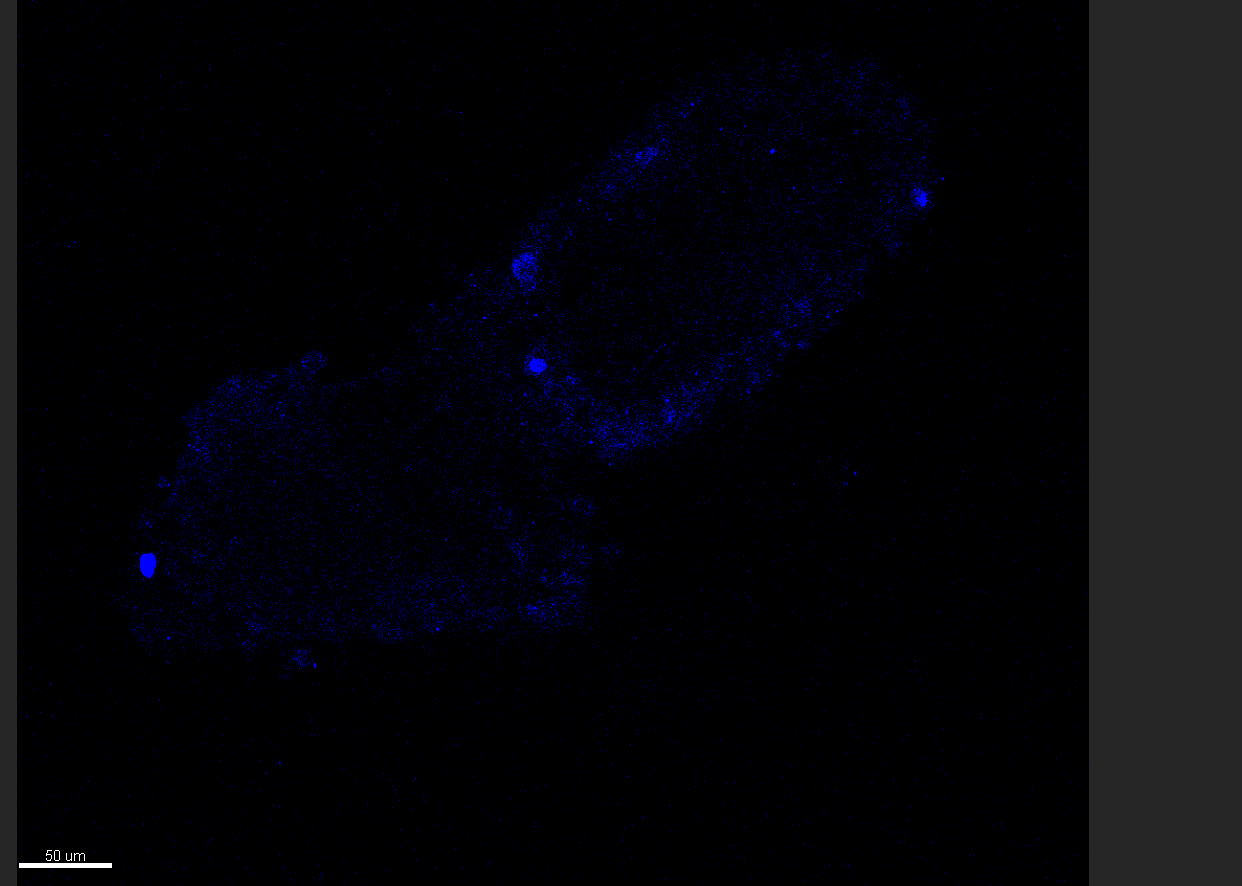

Supplement: Supplementary file 13 — Source data Fig. 1 [file 44318_2025_417_MOESM13_ESM.zip › Figure 1/Figure 1F/Serum:LIF_GATA6.tif]

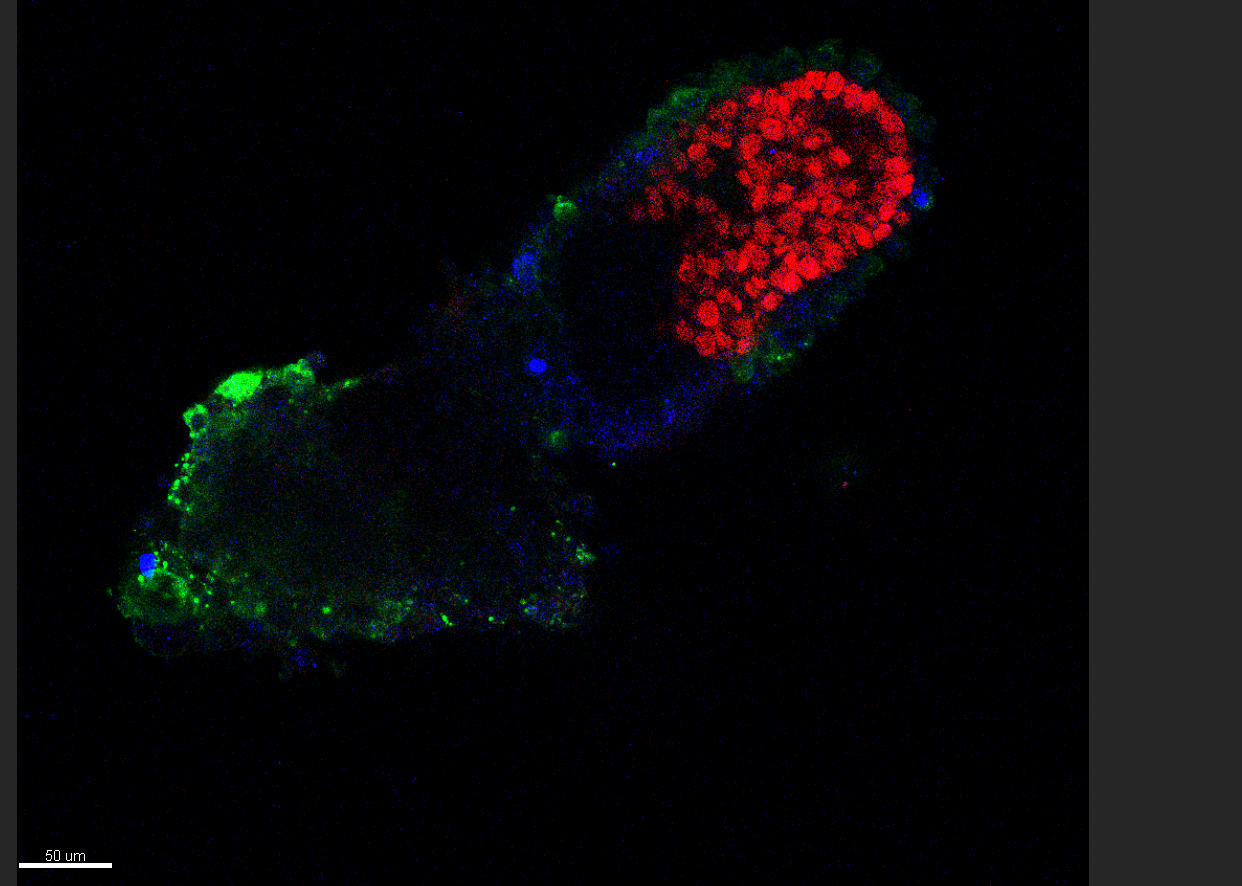

Supplement: Supplementary file 13 — Source data Fig. 1 [file 44318_2025_417_MOESM13_ESM.zip › Figure 1/Figure 1F/Serum:LIF_Merge.tif]

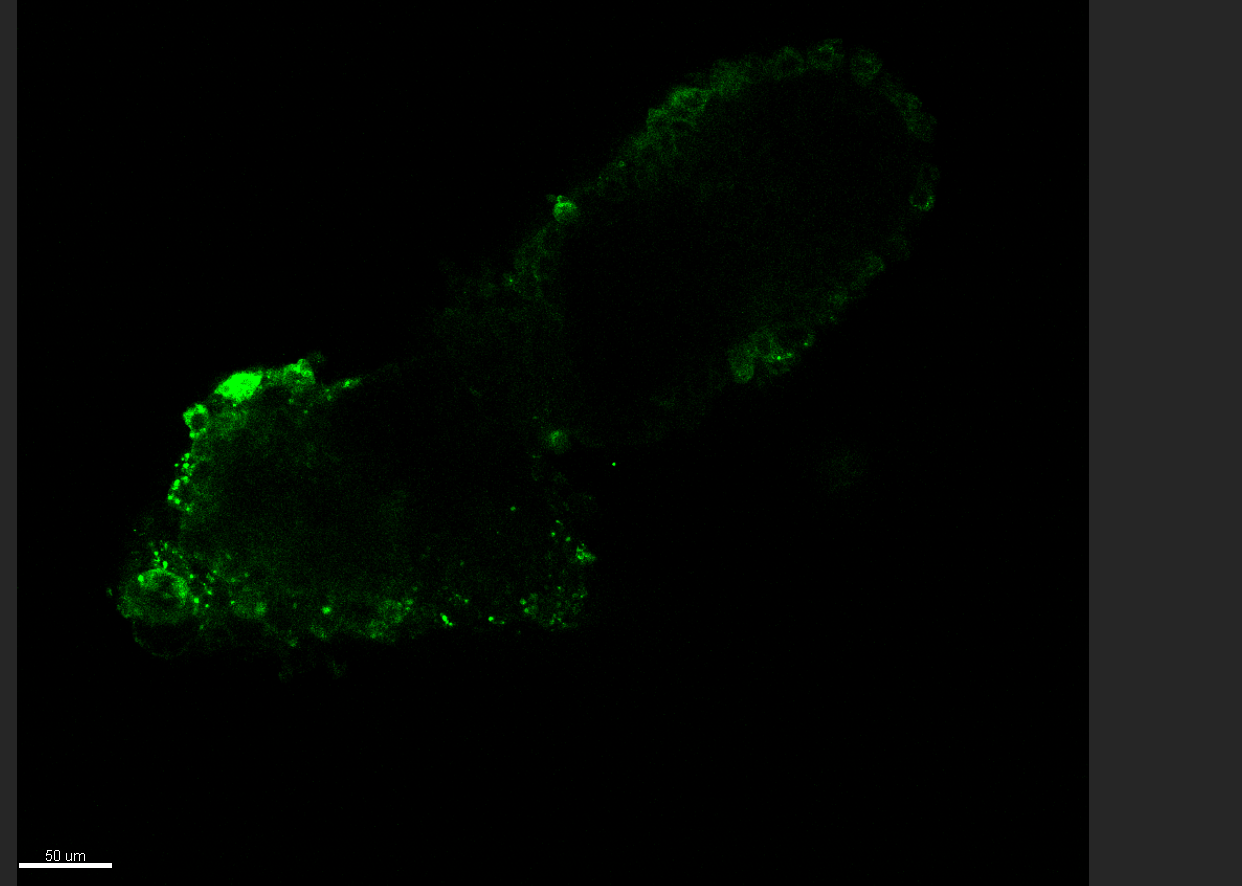

Supplement: Supplementary file 13 — Source data Fig. 1 [file 44318_2025_417_MOESM13_ESM.zip › Figure 1/Figure 1F/Serum:LIF_KRT7.tif]

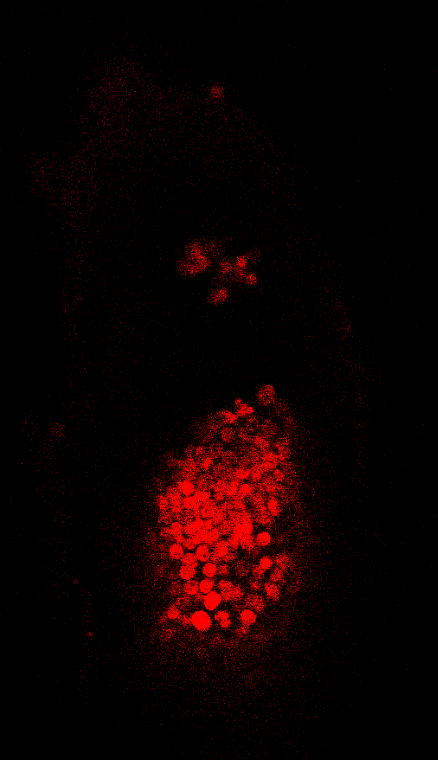

Supplement: Supplementary file 13 — Source data Fig. 1 [file 44318_2025_417_MOESM13_ESM.zip › Figure 1/Figure 1F/EMM_H2B-Tomato.tif]

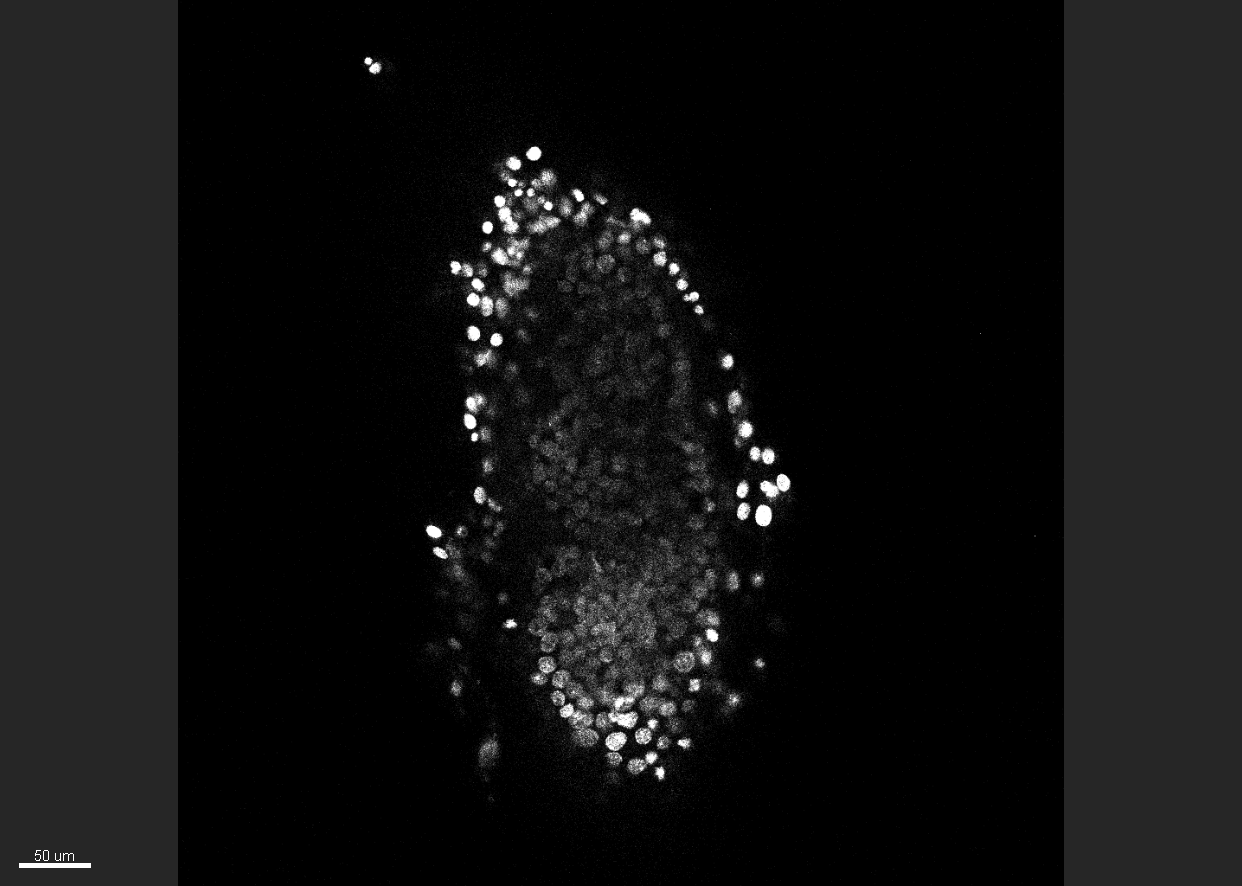

Supplement: Supplementary file 13 — Source data Fig. 1 [file 44318_2025_417_MOESM13_ESM.zip › Figure 1/Figure 1F/EMM_DAPI.tif]

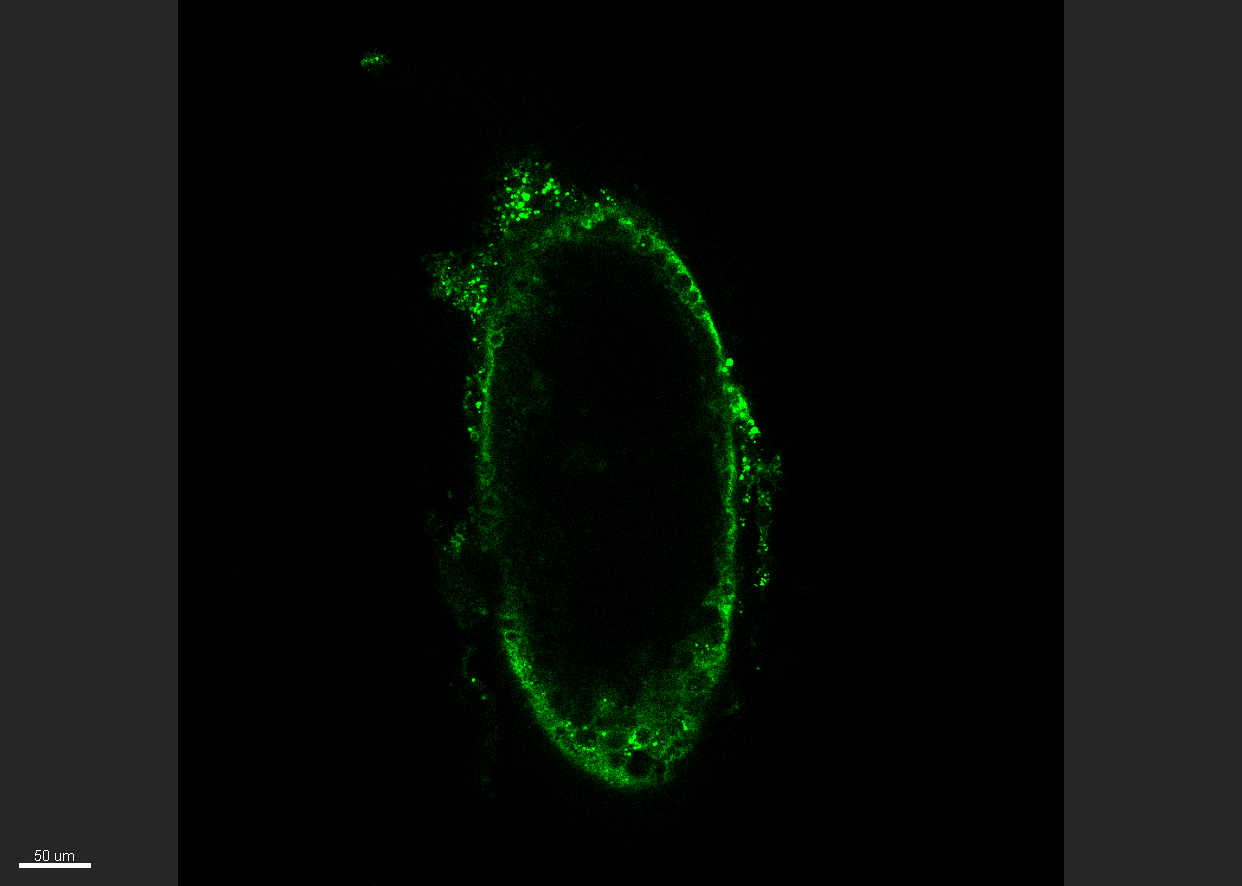

Supplement: Supplementary file 13 — Source data Fig. 1 [file 44318_2025_417_MOESM13_ESM.zip › Figure 1/Figure 1F/EMM_KRT7.tif]

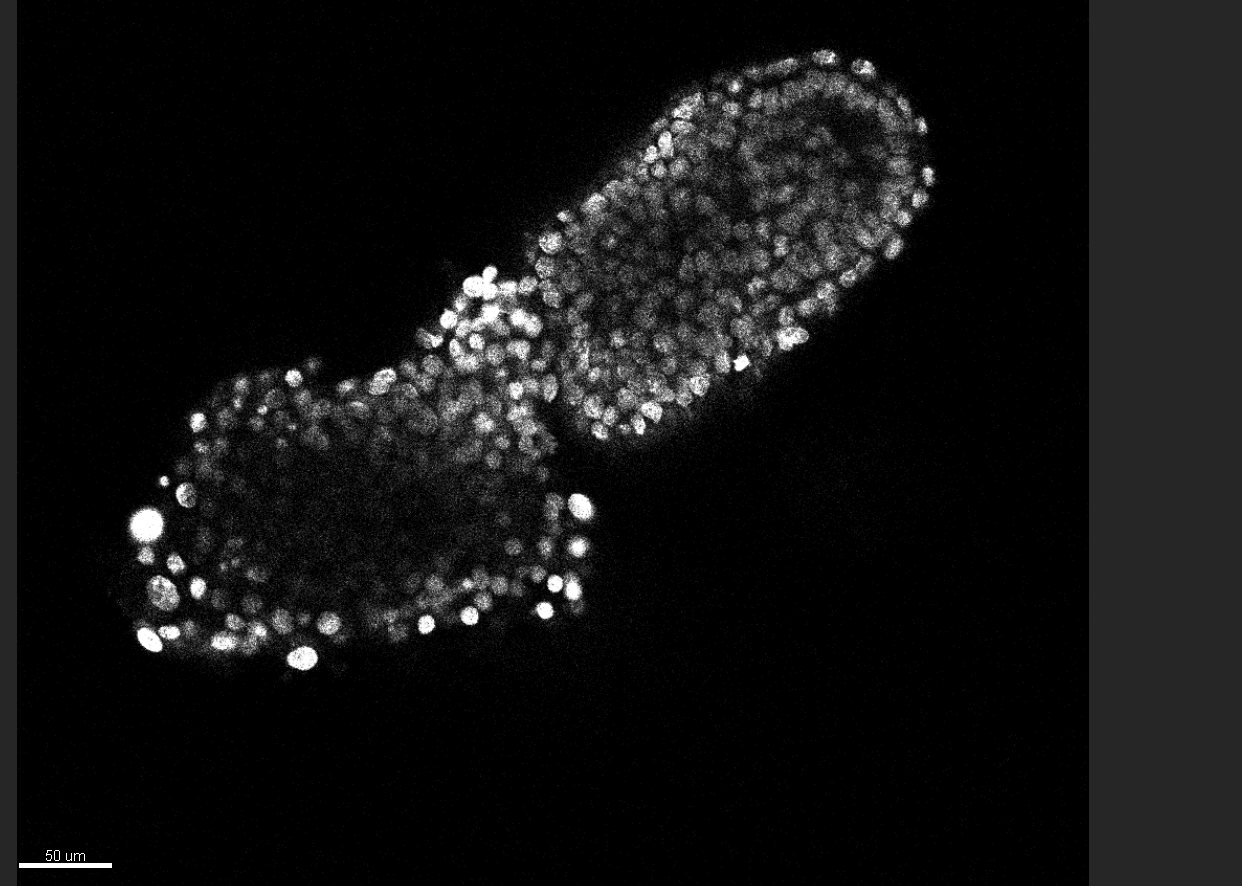

Supplement: Supplementary file 13 — Source data Fig. 1 [file 44318_2025_417_MOESM13_ESM.zip › Figure 1/Figure 1F/Serum:LIF_DAPI.tif]

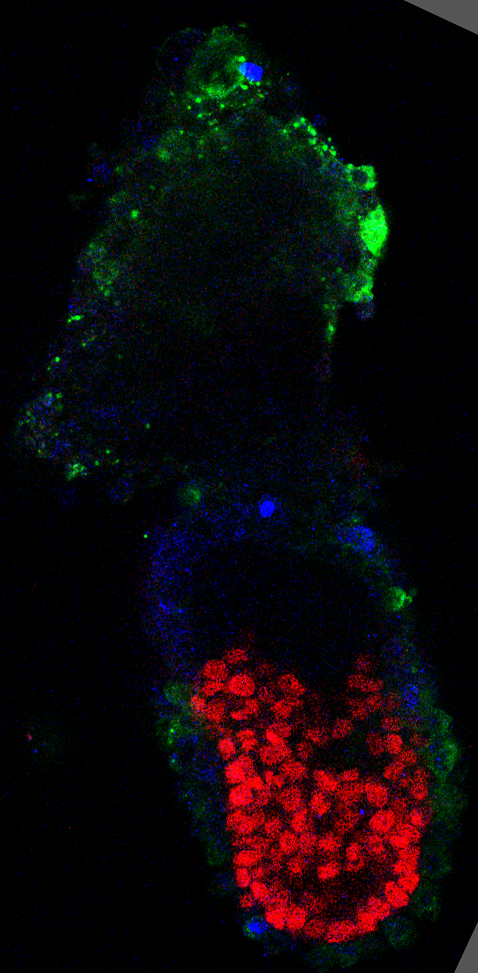

Supplement: Supplementary file 13 — Source data Fig. 1 [file 44318_2025_417_MOESM13_ESM.zip › Figure 1/Figure 1F/Serum:LIF_Merge_ps.tif]

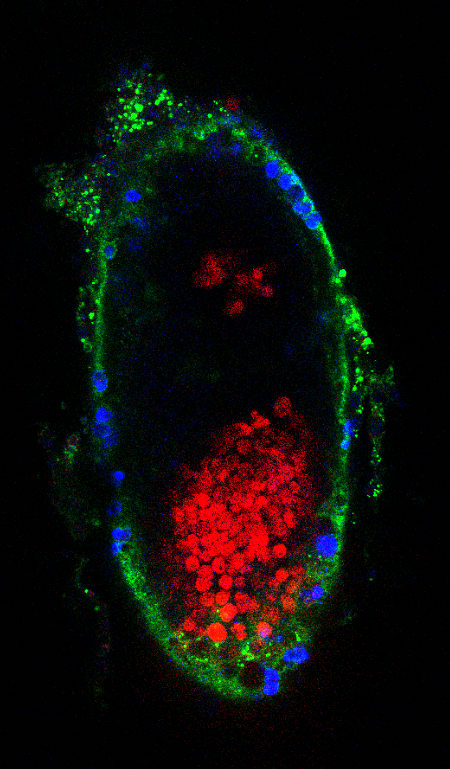

Supplement: Supplementary file 13 — Source data Fig. 1 [file 44318_2025_417_MOESM13_ESM.zip › Figure 1/Figure 1F/EMM_Merge_ps.tif]

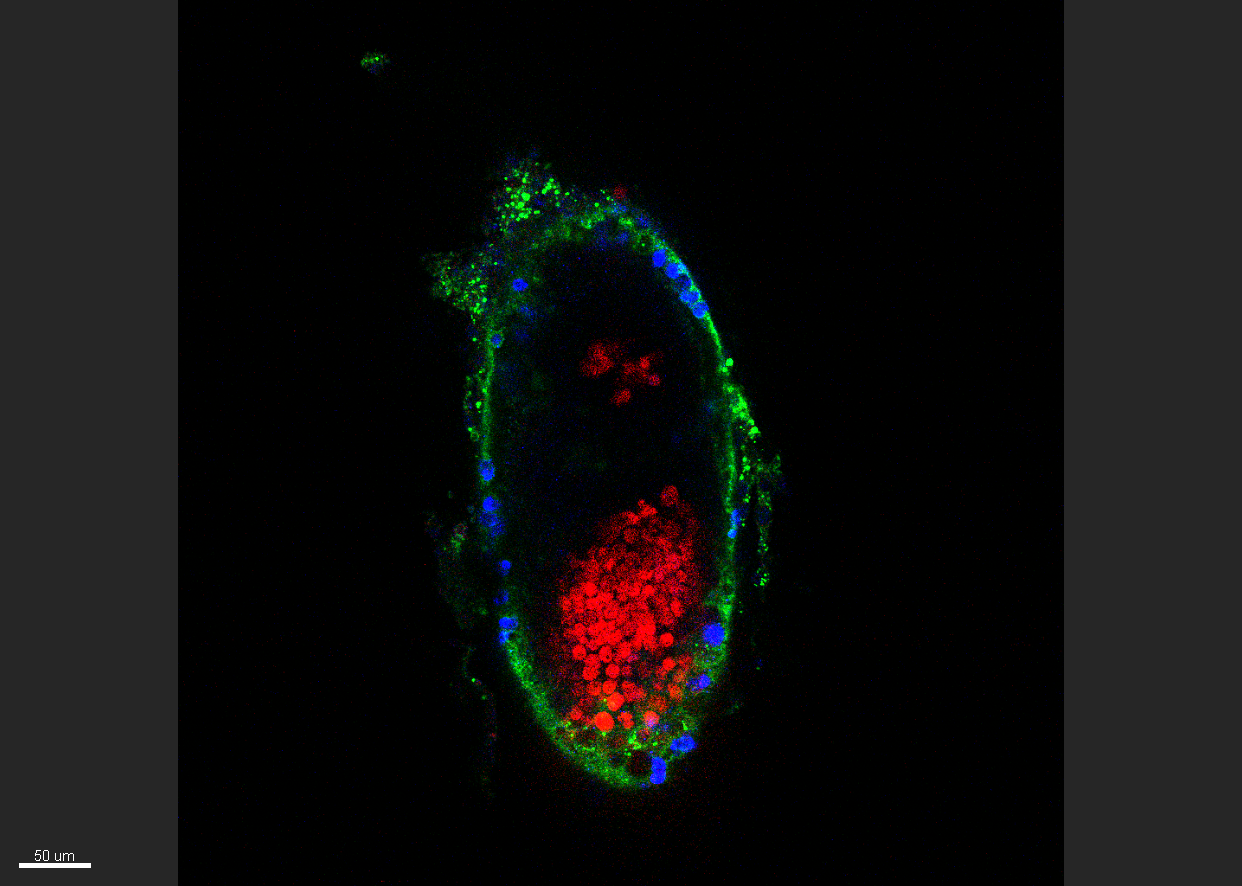

Supplement: Supplementary file 13 — Source data Fig. 1 [file 44318_2025_417_MOESM13_ESM.zip › Figure 1/Figure 1F/EMM_Merge.tif]

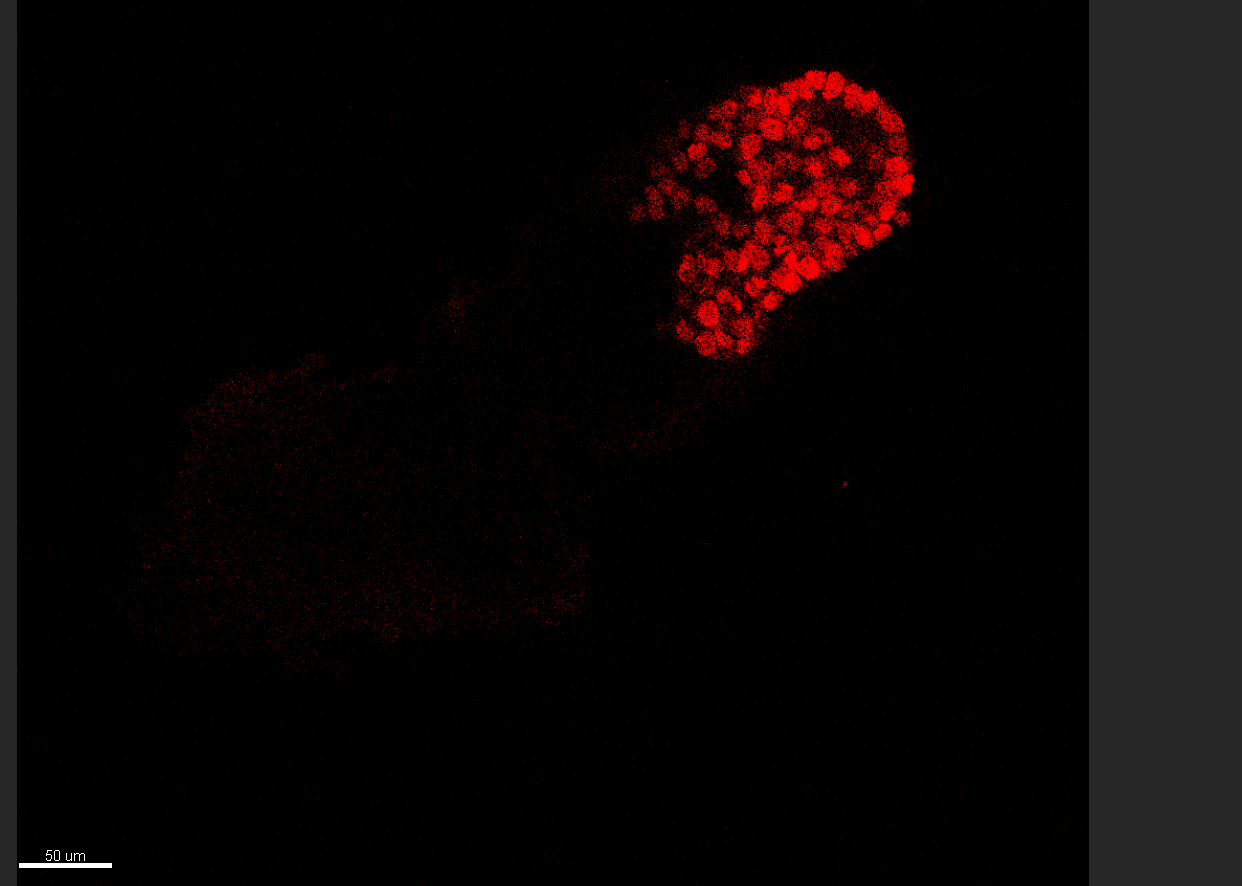

Supplement: Supplementary file 13 — Source data Fig. 1 [file 44318_2025_417_MOESM13_ESM.zip › Figure 1/Figure 1F/Serum:LIF_H2B-Tomato.tif]

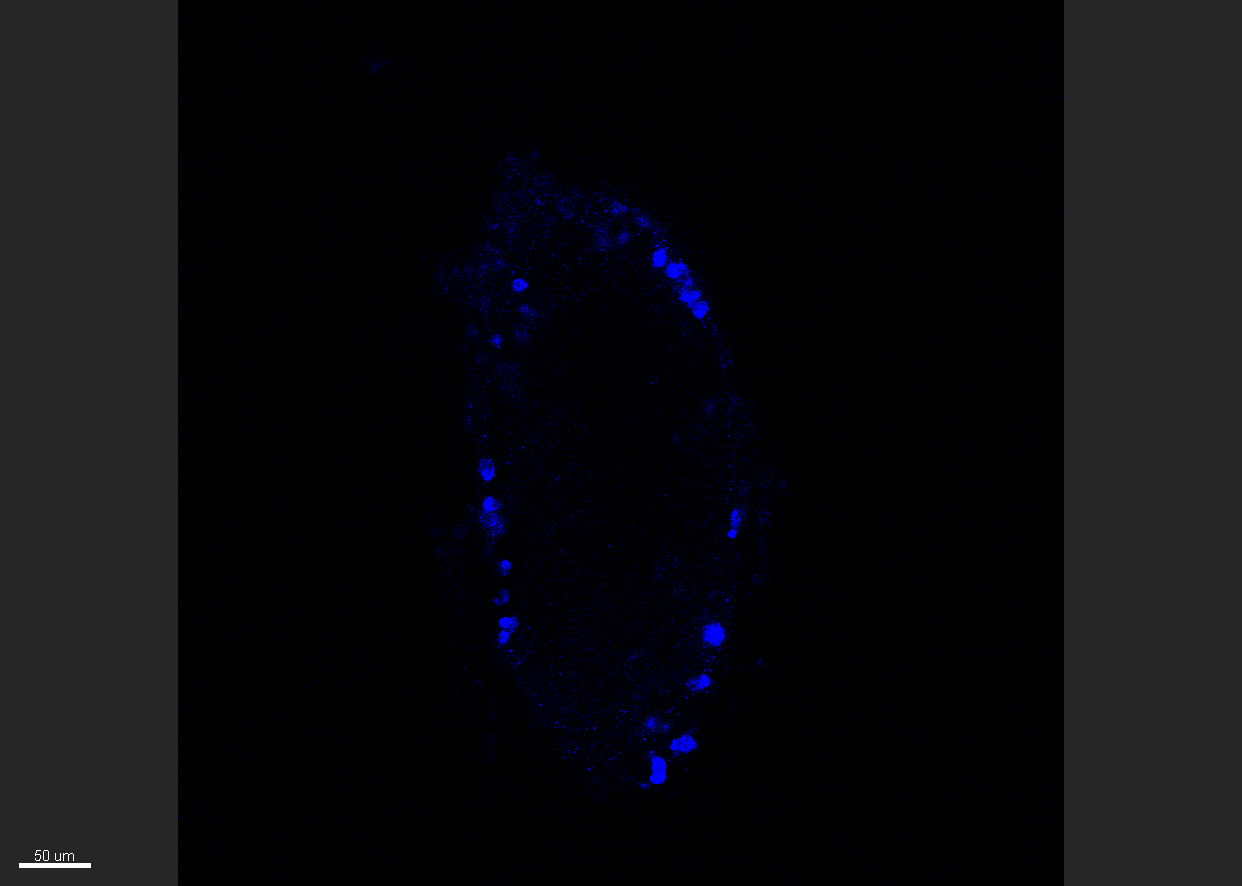

Supplement: Supplementary file 13 — Source data Fig. 1 [file 44318_2025_417_MOESM13_ESM.zip › Figure 1/Figure 1F/EMM_GATA6.tif]

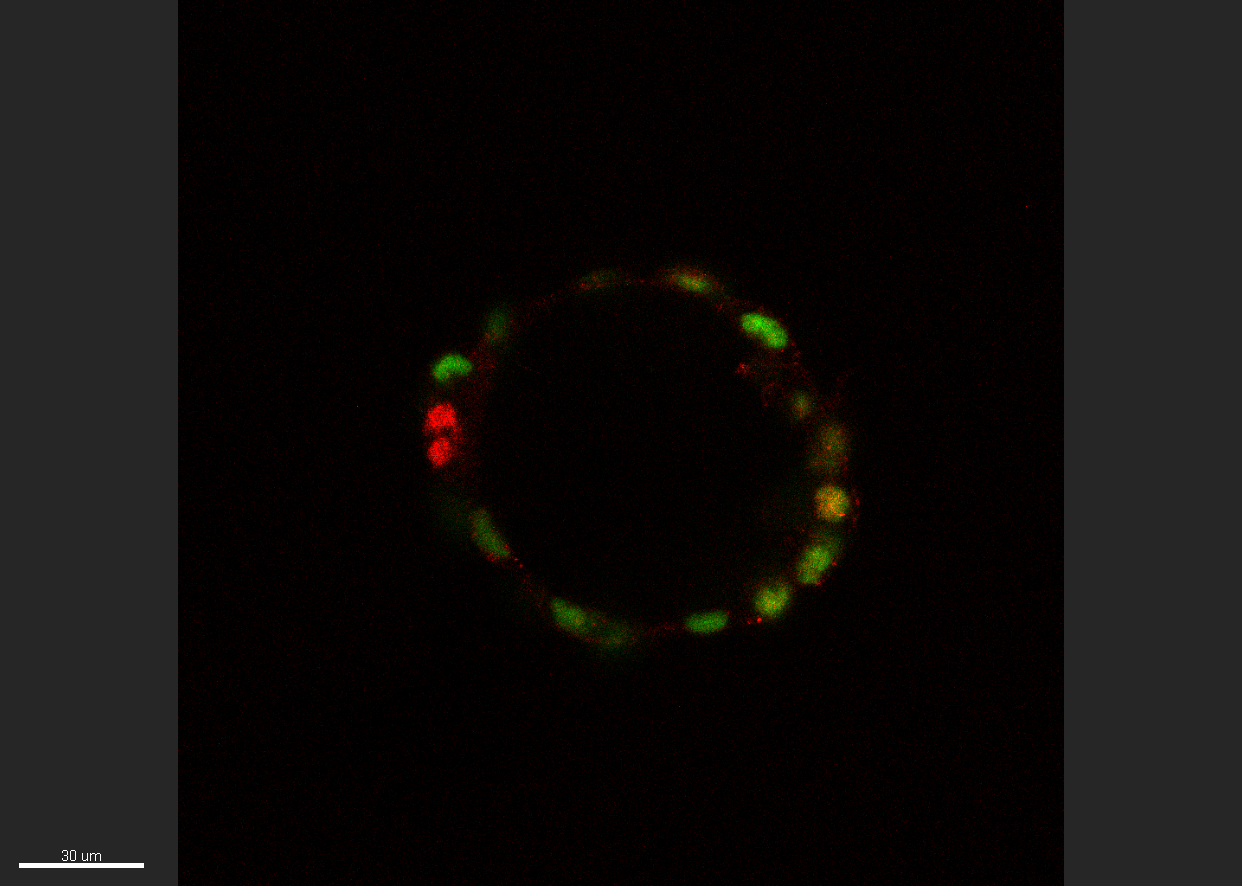

Supplement: Supplementary file 13 — Source data Fig. 1 [file 44318_2025_417_MOESM13_ESM.zip › Figure 1/Figure 1E/Serum:LIF_Merge.tif]

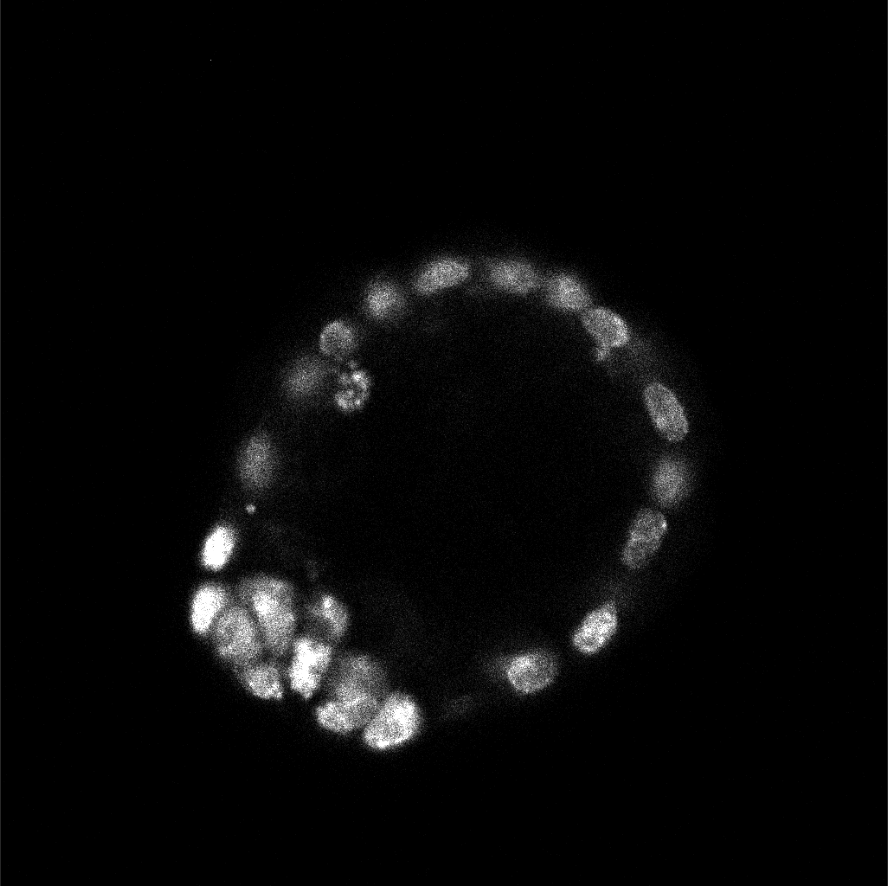

Supplement: Supplementary file 13 — Source data Fig. 1 [file 44318_2025_417_MOESM13_ESM.zip › Figure 1/Figure 1E/EMM_DAPI.tif]

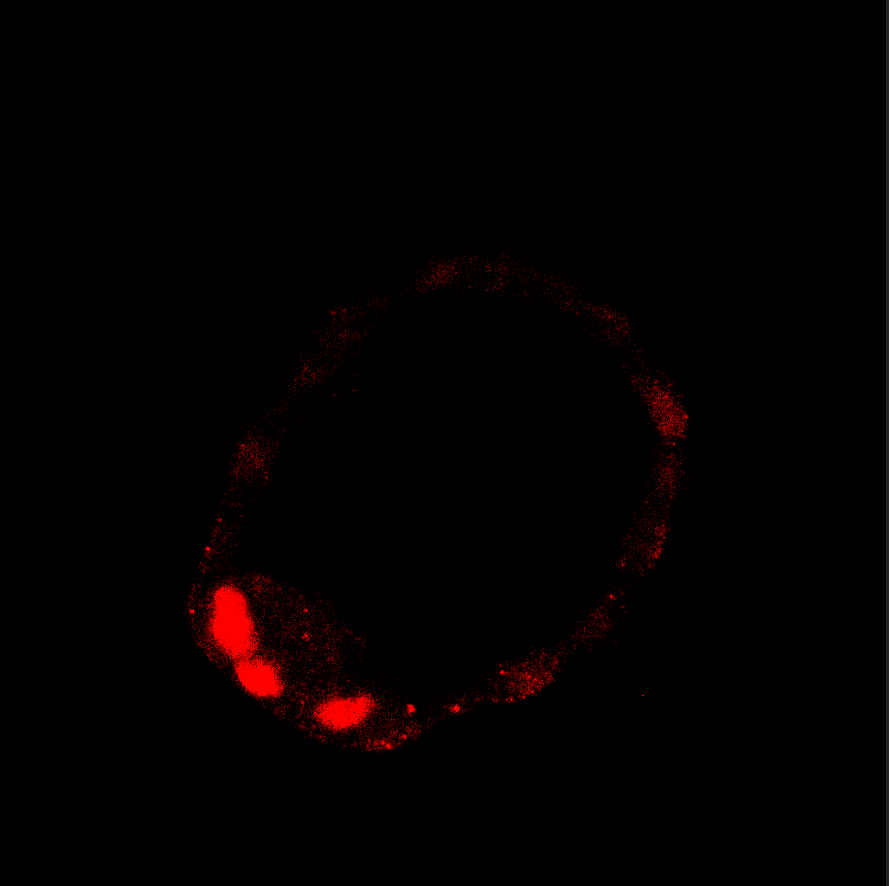

Supplement: Supplementary file 13 — Source data Fig. 1 [file 44318_2025_417_MOESM13_ESM.zip › Figure 1/Figure 1E/EMM_NANOG.tif]

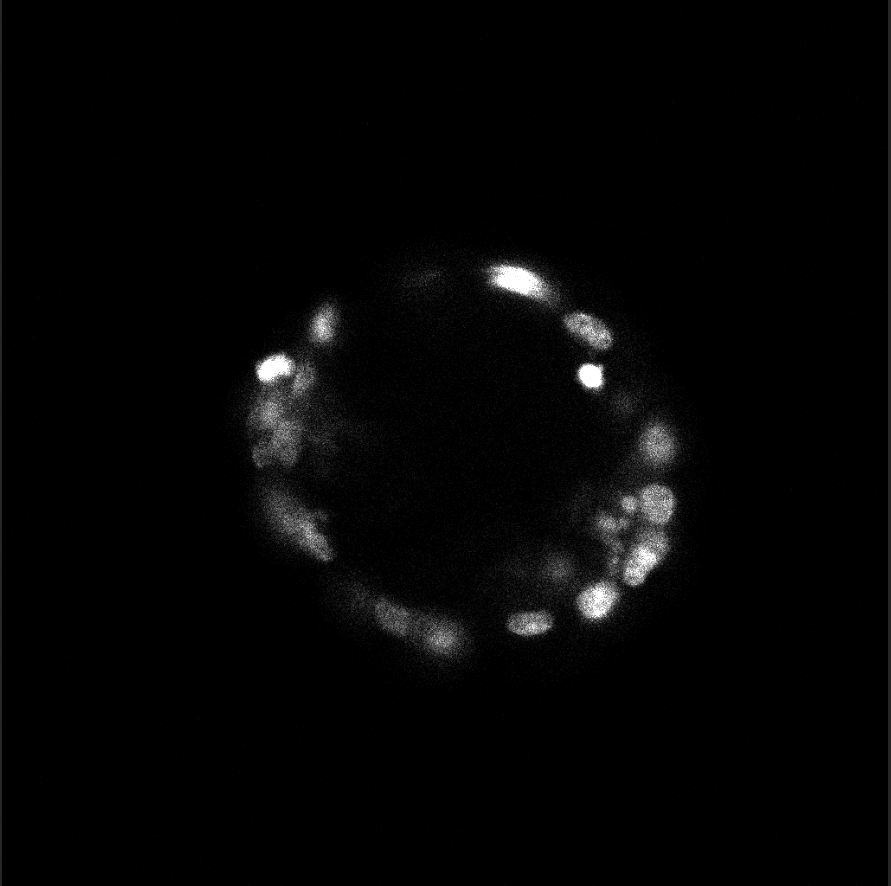

Supplement: Supplementary file 13 — Source data Fig. 1 [file 44318_2025_417_MOESM13_ESM.zip › Figure 1/Figure 1E/Serum:LIF_DAPI.tif]

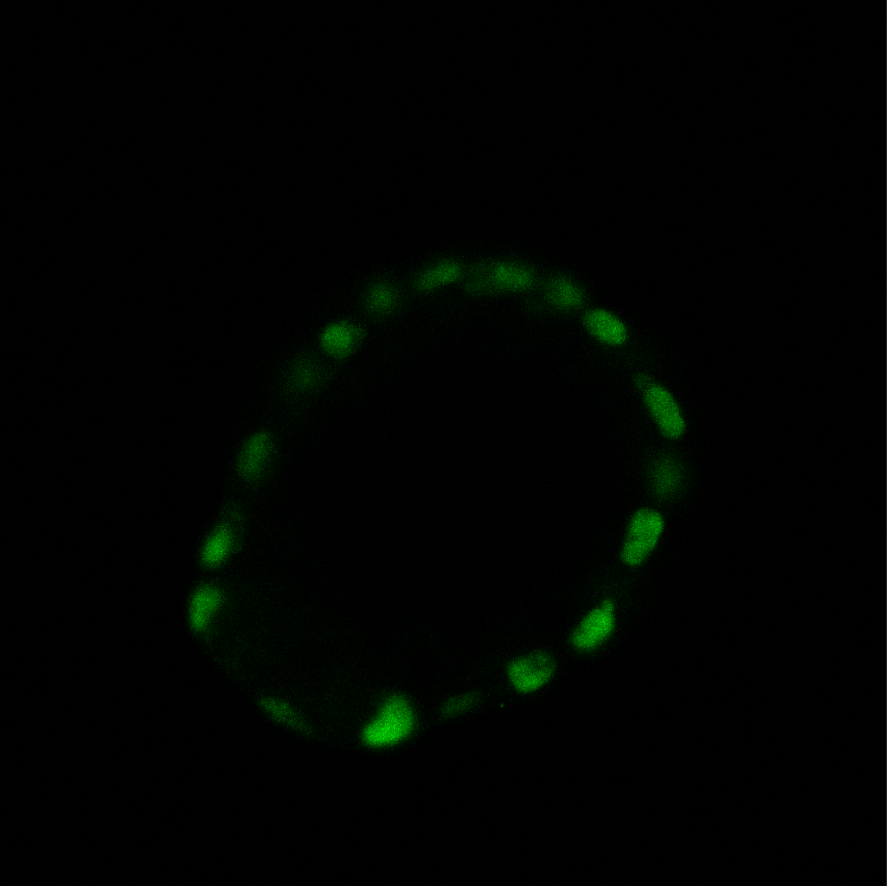

Supplement: Supplementary file 13 — Source data Fig. 1 [file 44318_2025_417_MOESM13_ESM.zip › Figure 1/Figure 1E/EMM_CDX2.tif]

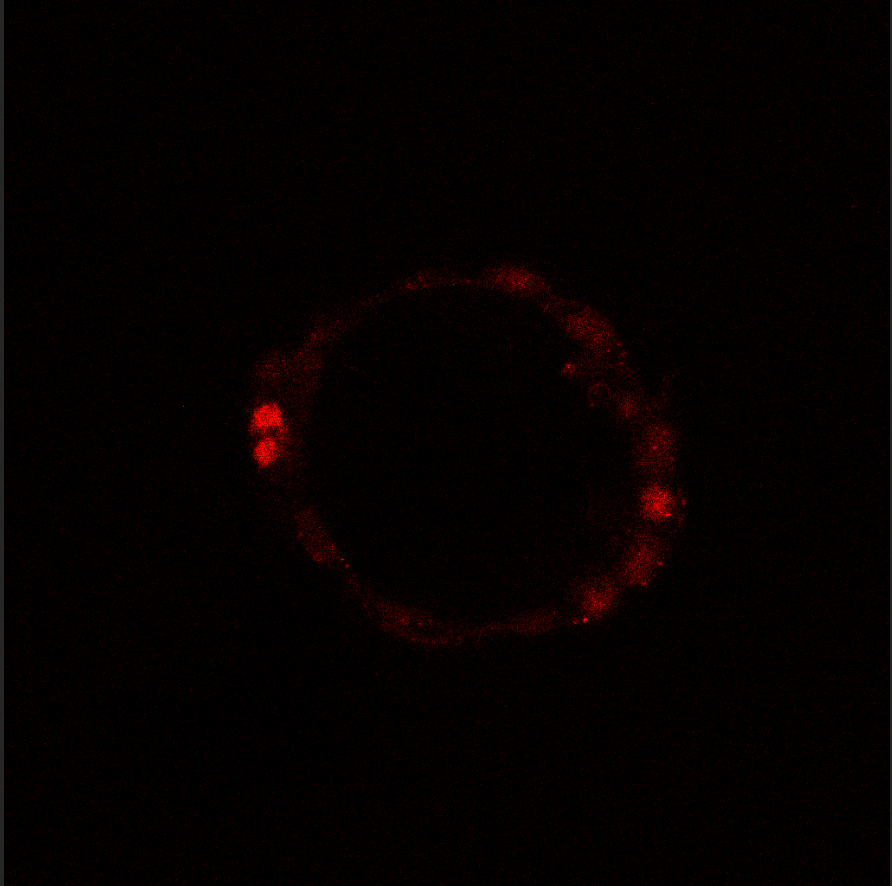

Supplement: Supplementary file 13 — Source data Fig. 1 [file 44318_2025_417_MOESM13_ESM.zip › Figure 1/Figure 1E/Serum:LIF_NANOG.tif]

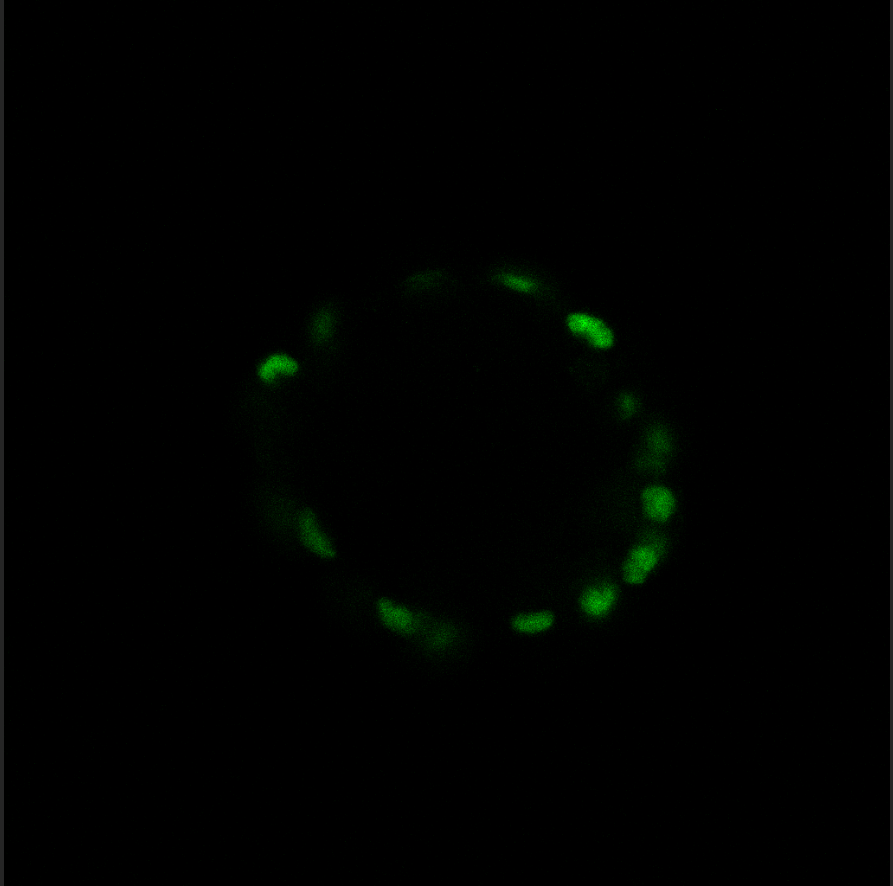

Supplement: Supplementary file 13 — Source data Fig. 1 [file 44318_2025_417_MOESM13_ESM.zip › Figure 1/Figure 1E/Serum:LIF_CDX2.tif]

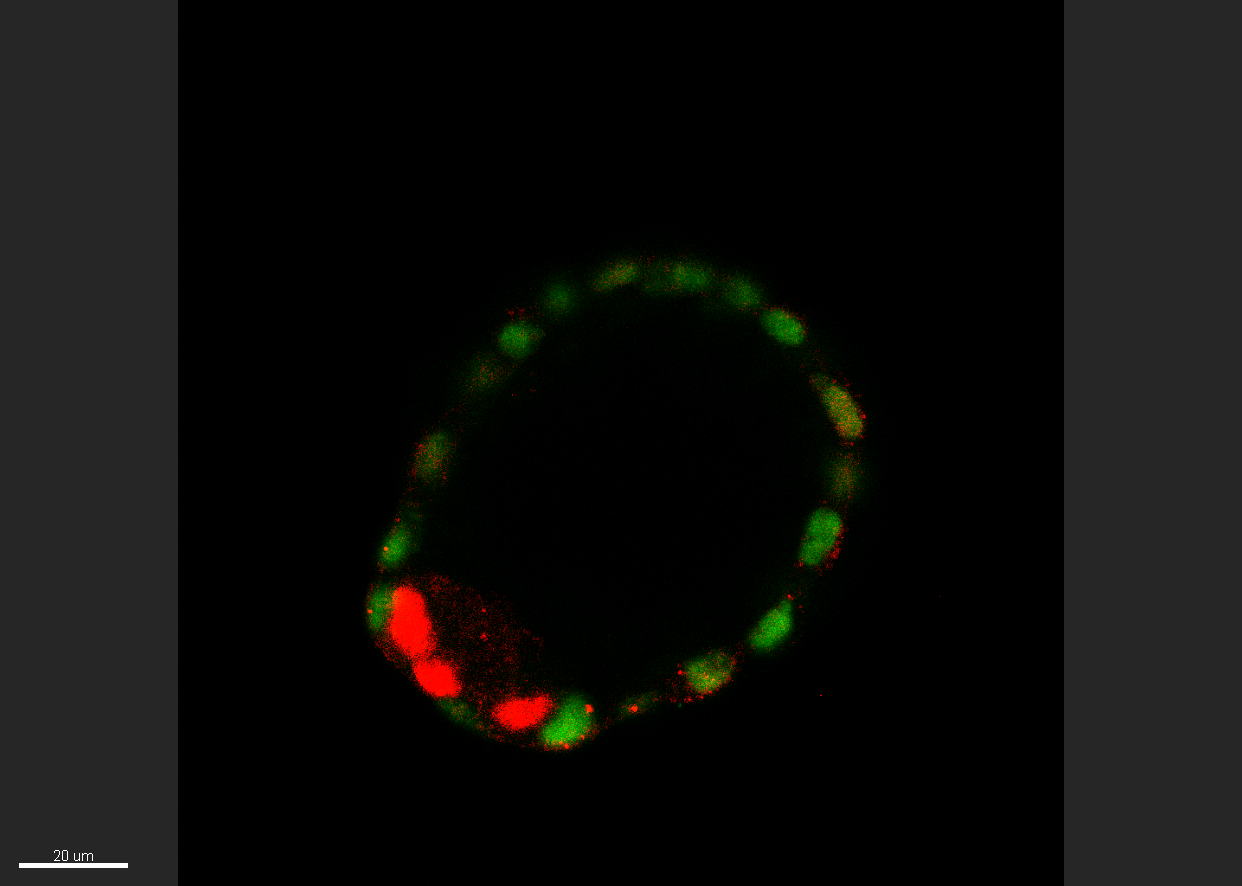

Supplement: Supplementary file 13 — Source data Fig. 1 [file 44318_2025_417_MOESM13_ESM.zip › Figure 1/Figure 1E/EMM_Merge.tif]

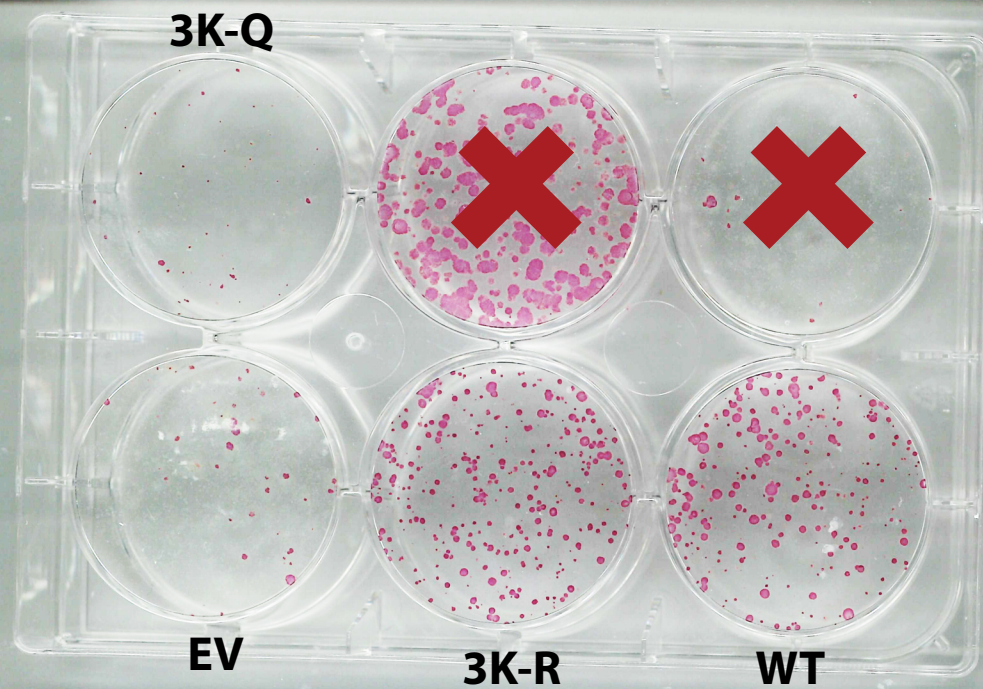

Supplement: Supplementary file 15 — Source data Fig. 4 [file 44318_2025_417_MOESM15_ESM.zip › Figure 4/AP SL.pdf]

**3K-Q**

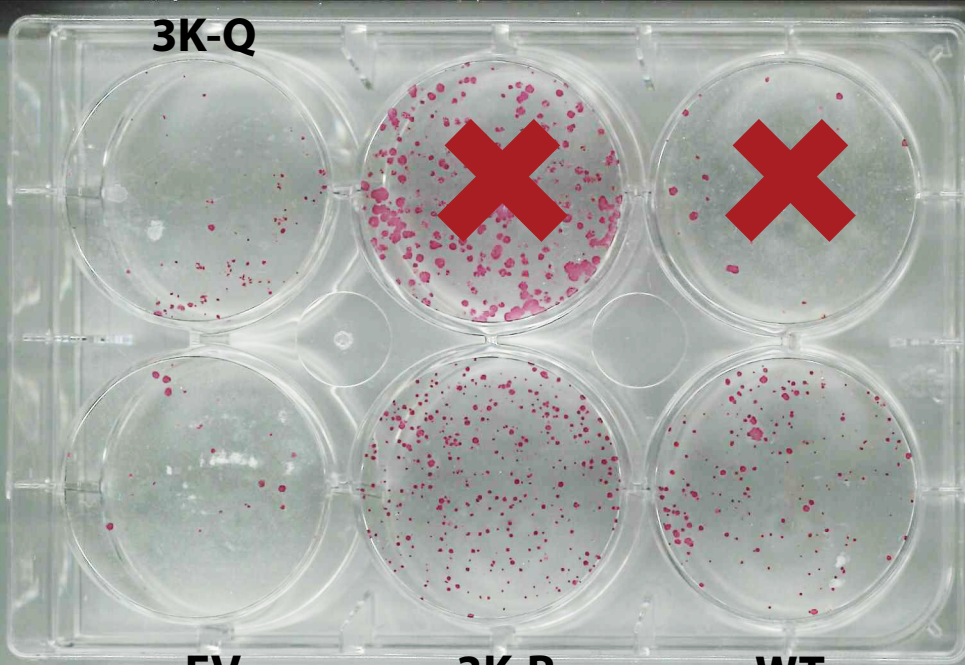

**EV**

**3K-R**

**WT**

Supplement: Supplementary file 15 — Source data Fig. 4 [file 44318_2025_417_MOESM15_ESM.zip › Figure 4/AP EMM.pdf]
