# Supplementary material for: Altering metabolism programs cell identity via NAD+-dependent deacetylation
Source: EMBO J. 2025 Apr 25;44(11):3056–84. doi: 10.1038/s44318-025-00417-0 (PMC12130289; doi:10.1038/s44318-025-00417-0)
Supplement: Supplementary file 16 — Expanded View Figures [file 44318_2025_417_MOESM16_ESM.pdf]

Expanded View Figures

Figure EV1. Differentiation potential characteristics of EMESCs and effects of galactose on pre-implantation development.

(**A**) Western blot analysis of SL-, EMM- and 2i/LIF-cultured mESCs. $n = 6$ biologically independent samples. Data are mean + s.d., unpaired two-tailed *t* test. *P* values: Active B-CATENIN SL vs EMM n.s.=0.8464, EMM vs 2i/LIF ***P* = 0.0040; pYAP1 SL vs EMM **P* = 0.0124, EMM vs 2i/LIF n.s.=0.0559; pERK SL vs EMM *****P* < 0.0001, EMM vs 2i/LIF *****P* < 0.0001; pAKT SL vs EMM *****P* < 0.0001, EMM vs 2i/LIF **P* = 0.0148; pSTAT3 SL vs EMM ***P* = 0.0069, EMM vs 2i/LIF n.s. *P* = 0.7369. (**B**) RT-qPCR analysis of pluripotency genes *Oct4*, *Nanog* and *Tfcp2l1*; PrE genes *Gata4*, *Sox7* and *cMyc*; and TE genes *Gata3* and *Eomes*. $n = 3$ biologically independent samples. Data are mean + s.d., unpaired two-tailed *t* test. *P* values: *Oct4* n.s.=0.4303; *Nanog* n.s.= 0.0729; *Tcfp2l1* n.s.= 0.1515; *cMyc* ***P* = 0.0053; *Gata4* ***P* = 0.0097; *Sox7* **P* = 0.0159; *Eomes* **P* = 0.0297; *Gata3* **P* = 0.0380. (**C**) RT-qPCR analysis of pluripotency genes *Nanog* and *Oct4*, and neural genes *Nestin* and *Zfp521* at d0 and d7 of neural differentiation. ESCs and EMESCs were cultured in Serum/LIF or EMM respectively for 2 passages prior to d0. Data are mean + s.d., $n = 3$ biologically independent samples, unpaired two-tailed *t* test. *P* values: *Nestin* n.s.=0.2267; *Zfp521* n.s.= 0.1027. (**D**) RT-qPCR analysis of pluripotency gene *Oct4* and PrE genes *Gata4*, *Gata6* and *Pdgfra* at d0, d4 and d7 of PrE differentiation. ESCs and EMESCs were cultured in Serum/LIF or EMM respectively for 2 passages prior to d0. Data are mean + s.d., $n = 3$ biologically independent samples, unpaired two-tailed *t* test. *P* values: *Gata6* ***P* = 0.0084; *Pdgfra* ***P* = 0.0050. (**E**) RT-qPCR analysis of pluripotency genes *Nanog* and *Oct4*, and TSC genes *Gata3* and *Elf5* at d0 and d7 of TSC differentiation. ESCs and EMESCs were cultured in Serum/LIF or EMM respectively for 2 passages prior to d0. Data are mean + s.d., $n = 3$ biologically independent samples, unpaired two-tailed *t* test. *Gata3* ***P* = 0.0011; *Elf5* **P* = 0.0389. (**F**) Brightfield images of mESCs after 10 passages cultured in Serum/LIF, EMM, and 2i/LIF. Scale bar = 100 μm. (**G–I**) Comparitive expression of apoptosis (**G**), embryonic (**H**) and extra-embryonic (**I**) genes from transcriptomic data in mESCs cultured for 10 and 20 passages in Serum/LIF, EMM, and 2i/LIF. $n = 3$ biologically independent samples. Data are mean + s.d., unpaired two-tailed *t* test. *P* values: *Bad* SL vs 2i/LIF P20 ***P* = 0.0245, EMM vs 2i/LIF P20 *****P* < 0.0001; *Bak1* EMM vs 2i/LIF P10 ***P* = 0.0012, SL vs EMM P20 ***P* = 0.0012, SL vs 2i/LIF P20 ***P* = 0.0039, EMM vs 2i/LIF P20 *****P* < 0.0001; *Bid* SL vs 2i/LIF P10 *****P* < 0.0001, EMM vs 2i/LIF P10 *****P* < 0.0001, SL vs EMM P20 *****P* < 0.0001, SL vs 2i/LIF P20 *****P* < 0.0001; *Caspase3* EMM vs 2i/LIF P10 **P* = 0.03913, SL vs 2i/LIF P20 *****P*< 0.0001, EMM vs 2i/LIF P20 *****P* < 0.0001; *Caspase9* SL vs 2i/LIF P10 *****P* < 0.0001, EMM vs 2i/LIF P10 *****P* < 0.0001, SL vs 2i/LIF P20 *****P* < 0.0001, EMM vs 2i/LIF P20 *****P* < 0.0001; *Tnfrsfla* SL vs 2i/LIF P10 ****P* = 0.0001, EMM vs 2i/LIF P10 *****P* < 0.0001, SL vs 2i/LIF P20 *****P* < 0.0001, EMM vs 2i/LIF P20 *****P* < 0.0001; *Nanog* SL vs 2i/LIF P10 **P* = 0.0180, EMM vs 2i/LIF P10 ***P* = 0.0022, SL vs EMM P20 ****P* = 0.0007, SL vs 2i/LIF P20 ***P* = 0.0086, EMM vs 2i/LIF P20 *****P* < 0.0001; *Nr0b1* SL vs EMM P10 *****P* < 0.0001, EMM vs 2i/LIF P10 *****P* < 0.0001, SL vs 2i/LIF P20 *****P* < 0.0001, EMM vs 2i/LIF P20 *****P* < 0.0001; *Pou5f1* SL vs EMM P10 ****P* = 0.0009, EMM vs 2i/LIF P10 ****P* = 0.0002; *Sall4*, SL vs EMM P10 *****P* < 0.0001, SL vs 2i/LIF P10 ****P* = 0.0007, EMM vs 2i/LIF P10 *****P* < 0.0001, SL vs EMM P20 ***P* = 0.0050, EMM vs 2i/LIF P20 *****P* < 0.0001; *Sirt1* SL vs EMM P10 *****P* < 0.0001, EMM vs 2i/LIF P10 *****P* < 0.0001, SL vs EMM P20 *****P* < 0.0001, EMM vs 2i/LIF P20 *****P* < 0.0001; *Sox2* SL vs EMM P10 *****P* < 0.0001, EMM vs 2i/LIF P10 *****P* < 0.0001, SL vs EMM P20 *****P* = 0.0001, EMM vs 2i/LIF P20 *****P* = 0.0001; *Col4a1*, SL vs EMM P10 *****P* < 0.0001, SL vs 2i/LIF P10 *****P* < 0.0001, EMM vs 2i/LIF P10 *****P* < 0.0001, SL vs EMM P20 **p = 0.0013, EMM vs 2i/LIF P20 *****P* < 0.0001; *Col4a2*, SL vs EMM P10 ***P* = 0.0013, SL vs 2i/LIF P10 ****P* = 0.0006, EMM vs 2i/LIF P10 *****P* < 0.0001, SL vs 2i/LIF P20 ***P* = 0.0037, EMM vs 2i/LIF P20 ***P* = 0.0024; *Dusp4*, SL vs 2i/LIF P10 *****P* < 0.0001, EMM vs 2i/LIF P10 *****P* < 0.0001, SL vs EMM P20 *****P* < 0.0001, SL vs 2i/LIF P20 *****P* < 0.0001, EMM vs 2i/LIF P20 *****P* < 0.0001; *Gata6*, SL vs 2i/LIF P20 *****P* = 0.0081, EMM vs 2i/LIF P20 *****P* = 0.0037; *Lrp2*, SL vs EMM P10 **P* = 0.0131, SL vs 2i/LIF P10 *****P* < 0.0001, EMM vs 2i/LIF P10 **P* = 0.0113, SL vs EMM P20 *****P* < 0.0001, EMM vs 2i/LIF P20 *****P* < 0.0001; *Sox7*, SL vs EMM P10 ***P* = 0.0015, EMM vs 2i/LIF P10 ****P* = 0.0003, SL vs 2i/LIF P20 ***P* = 0.0099, EMM vs 2i/LIF P20 ****P* = 0.0006. (**J**) Representative image of mouse litter, star indicates mice with contribution (left); Table depicting contribution effeciency of mESCs (two different cell lines) cultured in SL or EMM in generating chimeric mice from injected blastocysts (right).

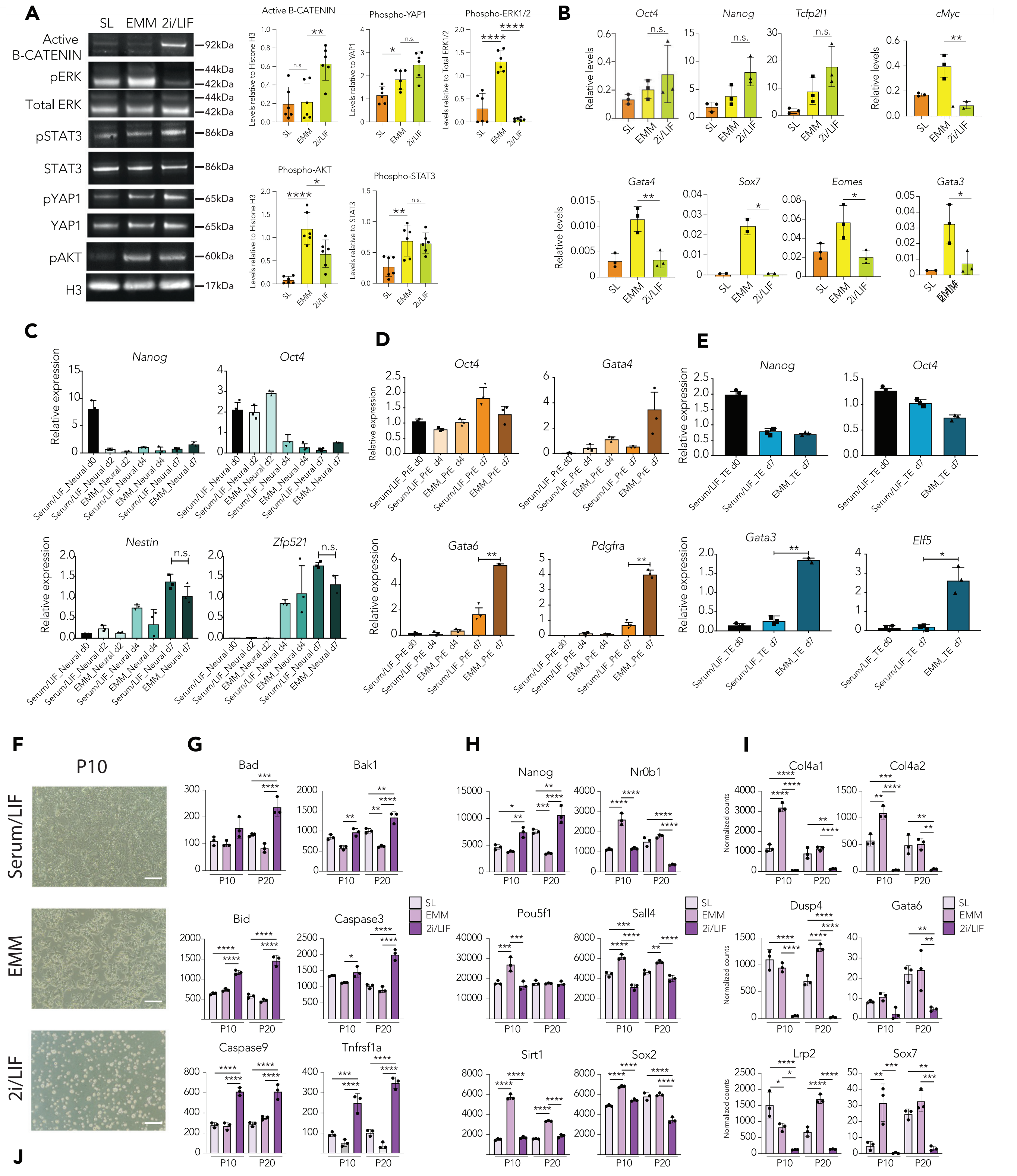

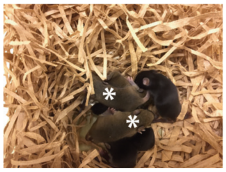

Cell line	Culture	Embryo number	Embryo stage	cells/embryo	pups born	chimeras	chimerism and gender
3.3	Glucose	20	Blastocyst	3	6	2	100%M and 70%M
		24	Blastocyst	3	3	1	100%M
	Galactose	36	Blastocyst	3	3	2	100%M and 90%M
3.4	Glucose	20	Blastocyst	3	3	0	
		36	Blastocyst	3	5	0	
	Galactose	36	Blastocyst	3	7	1	100%M

 © The Author(s)

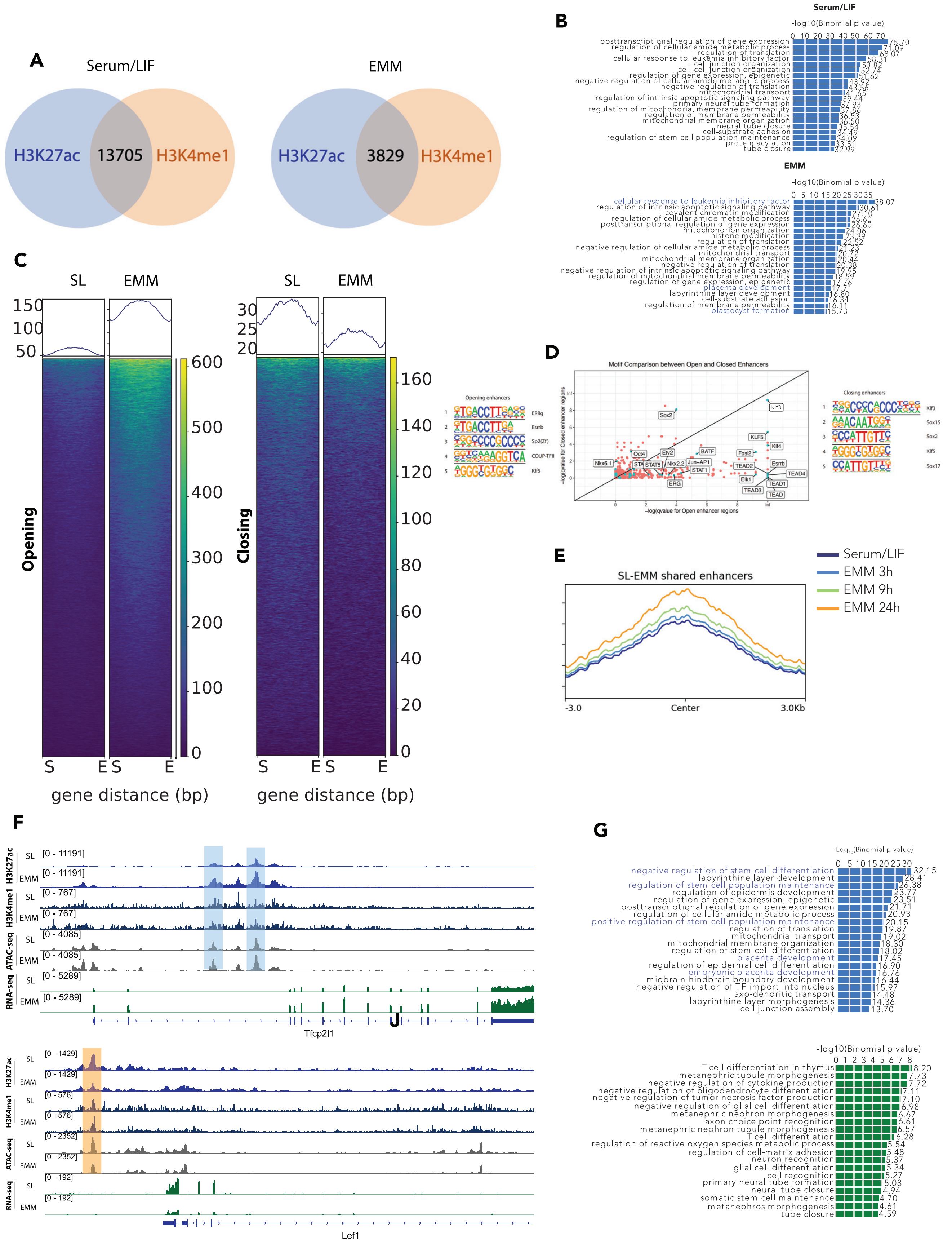
A
Serum/LIF
H3K27ac
13705
H3K4me1
EMM
H3K27ac
3829
H3K4me1
B
Serum/LIF
-log10(Binomial p value)
posttranscriptional regulation of gene expression 75.70
regulation of cellular amide metabolic process 71.09
regulation of translation 68.07
cellular response to leukemia inhibitory factor 58.31
cell junction organization 53.82
cell-cell junction organization 52.74
regulation of gene expression, epigenetic 51.62
negative regulation of cellular amide metabolic process 43.92
negative regulation of translation 43.56
mitochondrial transport 41.65
regulation of intrinsic apoptotic signaling pathway 39.44
primary neural tube formation 37.93
regulation of mitochondrial membrane permeability 37.86
regulation of membrane permeability 36.53
mitochondrial membrane organization 36.50
neural tube closure 35.54
cell-substrate adhesion 34.49
regulation of stem cell population maintenance 34.09
protein acylation 33.51
tube closure 32.99
EMM
-log10(Binomial p value)
cellular response to leukemia inhibitory factor 38.07
regulation of intrinsic apoptotic signaling pathway 30.61
covalent chromatin modification 27.10
regulation of cellular amide metabolic process 26.60
posttranscriptional regulation of gene expression 26.60
mitochondrion organization 24.06
histone modification 23.39
regulation of translation 22.52
negative regulation of cellular amide metabolic process 21.23
mitochondrial transport 20.72
mitochondrial membrane organization 20.44
negative regulation of translation 20.38
negative regulation of intrinsic apoptotic signaling pathway 19.95
regulation of mitochondrial membrane permeability 18.59
regulation of gene expression, epigenetic 17.76
placenta development 17.71
labyrinthine layer development 16.80
cell-substrate adhesion 16.34
regulation of membrane permeability 16.11
blastocyst formation 15.73
C
SL
EMM
Opening
Closing
gene distance (bp)
D
Motif Comparison between Open and Closed Enhancers
-log(qvalue for Open enhancer regions)
-log(qvalue for Closed enhancer regions)
Opening enhancers
ERRg
Esrrb
Sp2(Zf)
COUP-TFII
Klf5
Closing enhancers
Klf3
Sox15
Sox2
Klf5
Sox17
E
SL-EMM shared enhancers
Serum/LIF
EMM 3h
EMM 9h
EMM 24h
-3.0
Center
3.0Kb
F
H3K27ac
H3K4me1
ATAC-seq
RNA-seq
SL
EMM
[0 - 11191]
[0 - 767]
[0 - 4085]
[0 - 5289]
Tfcp2l1
G
-Log10(Binomial p value)
negative regulation of stem cell differentiation 32.15
labyrinthine layer development 28.41
regulation of stem cell population maintenance 26.38
regulation of epidermis development 23.77
regulation of gene expression, epigenetic 23.51
posttranscriptional regulation of gene expression 21.71
regulation of cellular amide metabolic process 20.93
positive regulation of stem cell population maintenance 20.15
regulation of translation 19.87
mitochondrial transport 19.02
mitochondrial membrane organization 18.30
regulation of stem cell differentiation 18.02
placenta development 17.45
regulation of epidermal cell differentiation 16.90
embryonic placenta development 16.76
midbrain-hindbrain boundary development 16.44
negative regulation of TF import into nucleus 15.97
axo-dendritic transport 14.48
labyrinthine layer morphogenesis 14.36
cell junction assembly 13.70
J
[0 - 1429]
[0 - 576]
[0 - 2352]
[0 - 192]
Lef1
-log10(Binomial p value)
T cell differentiation in thymus 18.20
metanephric tubule morphogenesis 7.73
negative regulation of cytokine production 7.72
negative regulation of oligodendrocyte differentiation 7.11
negative regulation of tumor necrosis factor production 7.10
negative regulation of glial cell differentiation 6.98
metanephric nephron morphogenesis 6.67
axon choice point recognition 6.61
metanephric nephron tubule morphogenesis 6.57
T cell differentiation 6.28
regulation of reactive oxygen species metabolic process 5.54
regulation of cell-matrix adhesion 5.48
neuron recognition 5.37
glial cell differentiation 5.34
cell recognition 5.27
primary neural tube formation 5.08
neural tube closure 4.94
somatic stem cell maintenance 4.70
metanephros morphogenesis 4.61
tube closure 4.59

© The Author(s)

Figure EV2. Further characterization of the enhancer landscape of EMM-cultured ESCs.

(**A**) Overlap of H3K27ac and H3K4me1 CUT&Tag peaks, defining enhancers in Serum/LIF and EMM. (**B**) GO analysis for Biological Processes of genes associated with enhancers defined by overlapping peaks for H3K27ac and H3K4me1 in ESCs cultured in Serum/LIF (top) and EMM (bottom). Discussed GO terms highlighted in blue. (**C**) Heatmaps and profiles depicting H3K27ac intensity in both Serum/LIF and EMM conditions at opening and closing enhancers. (**D**) Motif enrichment analysis of Opening versus Closing enhancers. (**E**) ATAC-seq time course metaprofiles of SL-EMM shared enhancers. (**F**) Genome browser tracks (IGV 2.14.0 software) of CUT&Tag for H3K27ac and H3K4me1, ATAC-seq and RNA-seq at the Tfcp2l1 locus (top) and Lef1 locus (bottom) at 0 h and 24 h of EMM treatment. Blue boxes depict opening enhancers, orange box depicts a closing enhancer. (**G**) GO analysis for Biological Processes of genes associated with ATAC-upregulated (top) and -downregulated (bottom) TE regions after 24 h EMM culture (noted processes in blue font).

 © The Author(s)

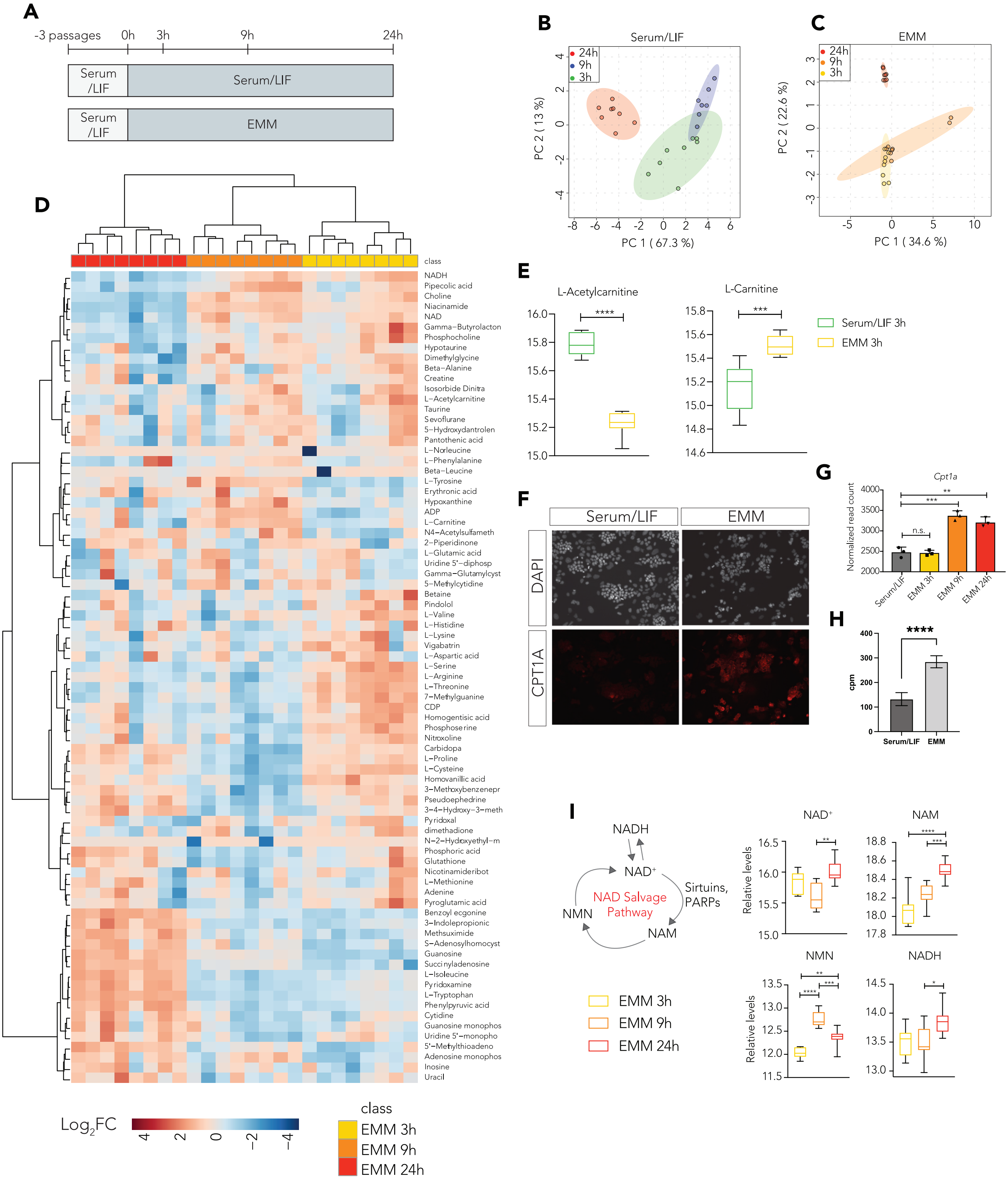
A
-3 passages
0h
3h
9h
24h
Serum /LIF
Serum/LIF
Serum /LIF
EMM
B
Serum/LIF
24h
9h
3h
PC 2 (13 %)
PC 1 (67.3 %)
C
EMM
24h
9h
3h
PC 2 (22.6 %)
PC 1 (34.6 %)
D
class
NADH
Pipecolic acid
Choline
Niacinamide
NAD
Gamma−Butyrolacton
Phosphocholine
Hypotaurine
Dimethylglycine
Beta−Alanine
Creatine
Isosorbide Dinitra
L−Acetylcarnitine
Taurine
Sevoflurane
5−Hydroxydantrolen
Pantothenic acid
L−Norleucine
L−Phenylalanine
Beta−Leucine
L−Tyrosine
Erythronic acid
Hypoxanthine
ADP
L−Carnitine
N4−Acetylsulfameth
2−Piperidinone
L−Glutamic acid
Uridine 5'−diphosp
Gamma−Glutamylcyst
5−Methylcytidine
Betaine
Pindolol
L−Valine
L−Histidine
L−Lysine
Vigabatrin
L−Aspartic acid
L−Serine
L−Arginine
L−Threonine
7−Methylguanine
CDP
Homogentisic acid
Phosphoserine
Nitroxoline
Carbidopa
L−Proline
L−Cysteine
Homovanillic acid
3−Methoxybenzenepr
Pseudoephedrine
3−4−Hydroxy−3−meth
Pyridoxal
dimethadione
N−2−Hydroxyethyl−m
Phosphoric acid
Glutathione
Nicotinamideribot
L−Methionine
Adenine
Pyroglutamic acid
Benzoyl ecgonine
3−Indolepropionic
Methsuximide
S−Adenosylhomocyst
Guanosine
Succinyladenosine
L−Isoleucine
Pyridoxamine
L−Tryptophan
Phenylpyruvic acid
Cytidine
Guanosine monophos
Uridine 5'−monopho
5'−Methylthioadeno
Adenosine monophos
Inosine
Uracil
Log2FC
class
EMM 3h
EMM 9h
EMM 24h
E
L-Acetylcarnitine
L-Carnitine
Serum/LIF 3h
EMM 3h
F
Serum/LIF
EMM
DAPI
CPT1A
G
Cpt1a
Normalized read count
Serum/LIF
EMM 3h
EMM 9h
EMM 24h
n.s.
H
cpm
Serum/LIF
EMM
I
NADH
NAD+
NAD Salvage Pathway
Sirtuins, PARPs
NMN
NAM
EMM 3h
EMM 9h
EMM 24h
NAD+
NAM
NMN
NADH
Relative levels

Figure EV3. Downstream responses to EMM metabolic phenotypes and the enzymes propagating them.

(**A**) Experimental outline for Serum/LIF and EMM time course used for metabolomic analysis. (**B**) PC1 vs 2 for Serum/LIF-cultured ESCs at 3 h, 9 h and 24 h in culture. (**C**) PC1 vs 2 for EMM-cultured ESCs at 3 h, 9 h and 24 h in culture. (**D**) Heatmap of enriched metabolites ($Log_2FC < 1$, $P_{adj} < 0.05$) in Serum/LIF-cultured ESCs versus all time points during EMM culture, $n = 8$ technical replicates per sample. (**E**) Levels of L-Acetylcarnitine (left) and L-Carnitine (right) in Serum/LIF and EMM-cultured ESCs after 3 h culture. P values: L-Acetylcarnitine ****$P < 0.0001$; L-Carnitine ***$P = 0.0004$, unpaired two-tailed t test, $n = 8$ technical replicates per sample. (**F**) Immunostaining for CPT1A in Serum/LIF and EMM-cultured ESCs after 24 h. Data are representative of 3 biological replicates. Scale bar = 40µm. (**G**) Normalized counts from RNA-seq for *Cpt1a* in Serum/LIF and EMM-cultured ESCs after 3 h, 9 h and 24 h in the indicated condition. P values: n.s.=0.846 ***$P = 0.0008$, **$P = 0.0015$, unpaired two-tailed t test, $n = 3$ biologically independent samples. (**H**) Fatty acid oxidation assay, measuring metabolism of C^{14}-labeled palmitic acid in SL- and EMM-cultured ESCs after 24 h, representative of 3 independent experiments. Data was normalized to protein levels. ****$P < 0.0001$, unpaired two-tailed t test. (**I**) Schematic for NAD^+ salvage pathway with individual plots for the depicted metabolites after 3 h, 9 h, 24 h in EMM culture. P values: NAD + 9 h vs 24 h **$P = 0.001$; NAM 3 h vs 24 h ****$P < 0.0001$, 9 h vs 24 h ***$P = 0.0003$; NMN 3 h vs 9 h ****$P < 0.001$, 3 h vs 24 h **$P = 0.0012$, 9 h vs 24 h ***$P = 0.0009$; NADH 9 h vs 24 h *$P = 0.0145$; unpaired two-tailed t test, $n = 8$ technical replicates per sample.

 © The Author(s)

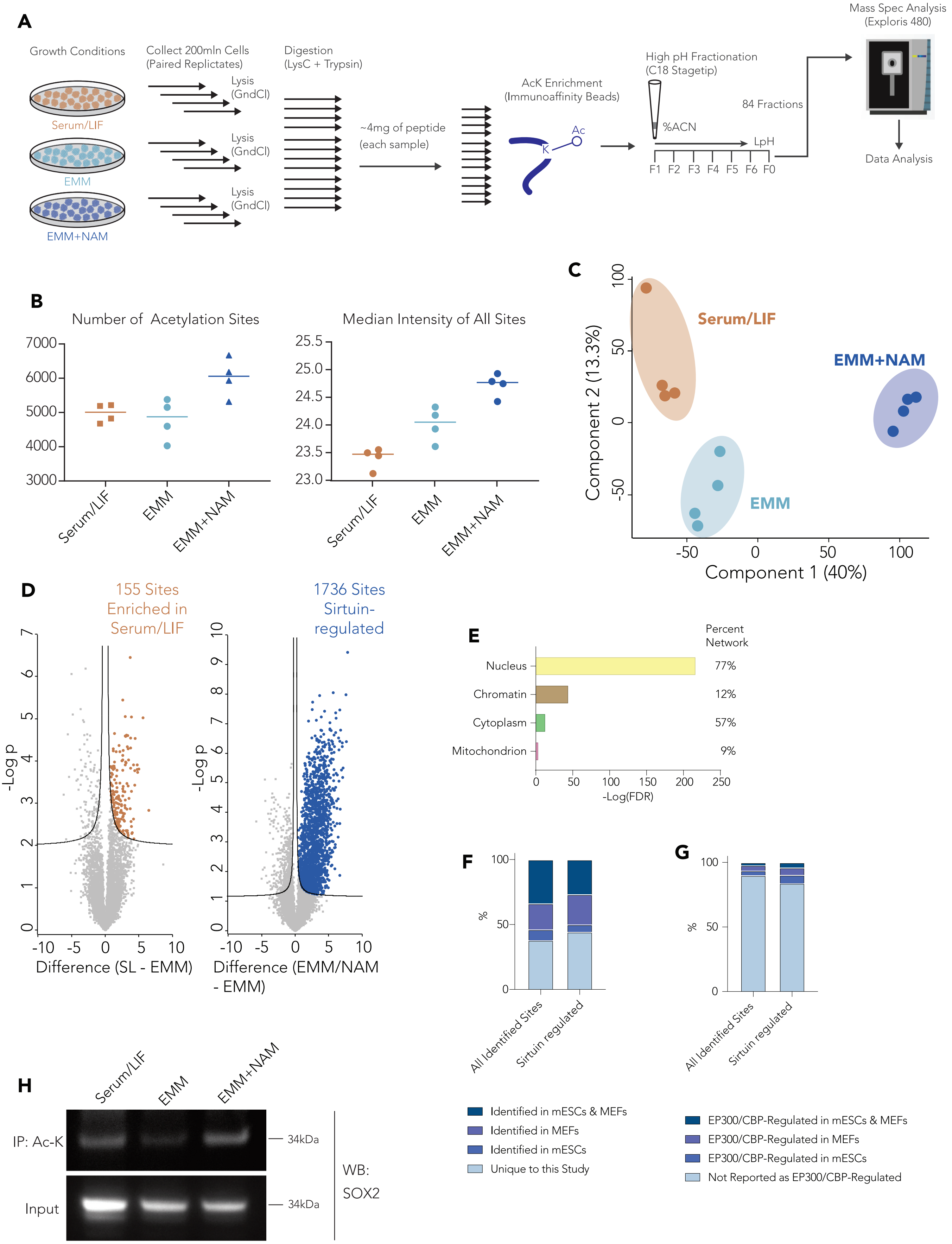
A
Growth Conditions
Serum/LIF
EMM
EMM+NAM
Collect 200mln Cells
(Paired Replictates)
Lysis
(GndCl)
Digestion
(LysC + Trypsin)
~4mg of peptide
(each sample)
AcK Enrichment
(Immunoaffinity Beads)
Ac
K
High pH Fractionation
(C18 Stagetip)
%ACN
LpH
84 Fractions
F1 F2 F3 F4 F5 F6 F0
Mass Spec Analysis
(Exploris 480)
Data Analysis
B
Number of Acetylation Sites
Median Intensity of All Sites
Serum/LIF
EMM
EMM+NAM
C
Serum/LIF
EMM+NAM
EMM
Component 2 (13.3%)
Component 1 (40%)
D
155 Sites
Enriched in
Serum/LIF
1736 Sites
Sirtuin-
regulated
-Log p
Difference (SL - EMM)
Difference (EMM/NAM
- EMM)
E
Percent
Network
Nucleus 77%
Chromatin 12%
Cytoplasm 57%
Mitochondrion 9%
-Log(FDR)
F
%
All Identified Sites
Sirtuin regulated
Identified in mESCs & MEFs
Identified in MEFs
Identified in mESCs
Unique to this Study
G
%
All Identified Sites
Sirtuin regulated
EP300/CBP-Regulated in mESCs & MEFs
EP300/CBP-Regulated in MEFs
EP300/CBP-Regulated in mESCs
Not Reported as EP300/CBP-Regulated
H
Serum/LIF
EMM
EMM+NAM
IP: Ac-K
Input
34kDa
34kDa
WB:
SOX2

Figure EV4. Further characterization of acetylome of EMM-cultured ESCs.

(**A**) The experimental setup to determine differential acetylation in ESCs cultured in Serum/LIF, EMM and EMM + NAM for 24 h. $n = 4$ experimentally independent samples. (**B**) Total number of acetylated sites detected in samples (left); Median intensity of all acetylated sites detected in samples (right). (**C**) PCA plot of acetylated sites in different samples. (**D**) Volcano plots depicting significantly differentially acetylated sites (FDR 5%) between EMM and Serum/LIF (left) and between EMM and EMM + NAM (right). (**E**) GO Cellular Component Enrichment table for sites deacetylated in EMM, with percent network. (**F**) Bar chart depicting percentage of all identified sites (6733) and that are also identified in datasets from (Weinert et al, 2018; Narita et al, 2021). (**G**) Bar chart depicting percentage of all identified sites (6733) and the overlap between the Sirtuin-regulated sites we defined (1731) and those regulated by EP300/CBP in datasets from (Weinert et al, 2018; Narita et al, 2021). (**H**) IP for Acetylated lysine and Western blot analysis of SOX2 in ESCs cultured for 24 h in Serum/LIF and EMM, +/− NAM. Data are representative of 3 biologically independent samples.

 © The Author(s)

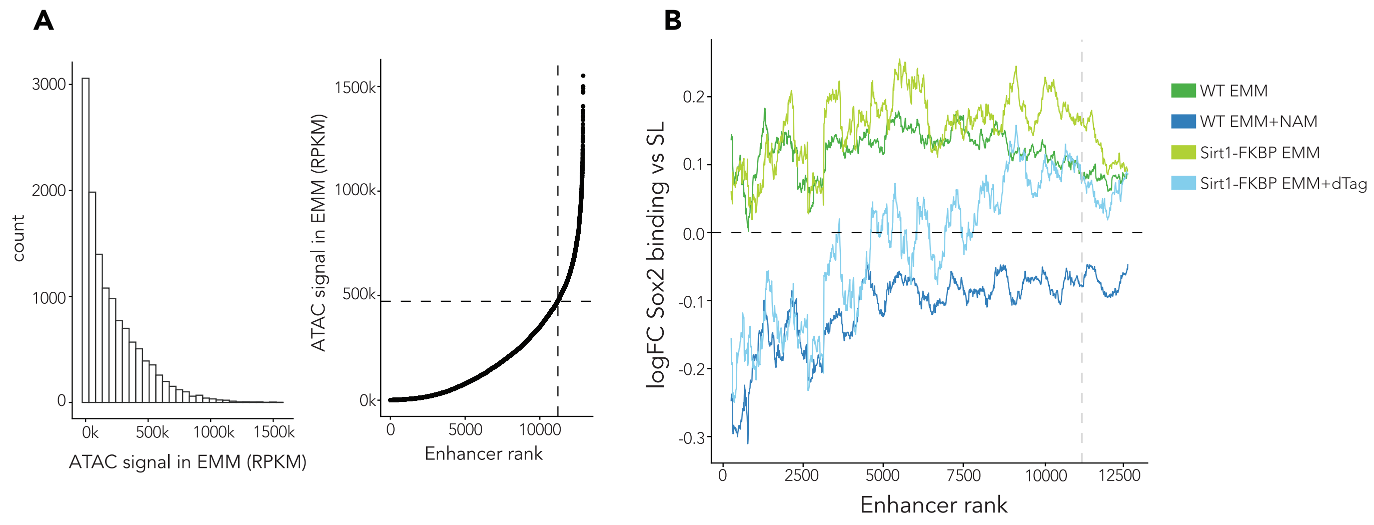

Figure EV5. Extra analysis of SIRT1 deacetylation of SOX2, and of SOX2 CUT&Tag in Sirt1-FKBP cell lines.

(**A**) Histogram ATAC signal count showing the number of SL-EMM merged enhancers plotted against ATAC signal in EMM (left), and enhancers ranked by ATAC signal (right). (**B**) Sliding window analysis of SOX2 signal pileup at enhancers ranked by ATAC signal in EMM, with the signal normalized to SL (for WT samples) and SL GFP+ (Sirt1-FKBP samples).
